# Diversity in gut bacterial community of school-age children in Asia

**DOI:** 10.1038/srep08397

**Published:** 2015-02-23

**Authors:** Jiro Nakayama, Koichi Watanabe, Jiahui Jiang, Kazunori Matsuda, Shiou-Huei Chao, Pri Haryono, Orawan La-ongkham, Martinus-Agus Sarwoko, I. Nengah Sujaya, Liang Zhao, Kang-Ting Chen, Yen-Po Chen, Hsueh-Hui Chiu, Tomoko Hidaka, Ning- Xin Huang, Chikako Kiyohara, Takashi Kurakawa, Naoshige Sakamoto, Kenji Sonomoto, Kousuke Tashiro, Hirokazu Tsuji, Ming-Ju Chen, Vichai Leelavatcharamas, Chii-Cherng Liao, Sunee Nitisinprasert, Endang S. Rahayu, Fa-Zheng Ren, Ying-Chieh Tsai, Yuan-Kun Lee

**Affiliations:** 1 Department of Bioscience and Biotechnology, Faculty of Agriculture, Kyushu University, 6-10-1 Hakozaki, Higashi-ku, Fukuoka 812-8581, Japan; 2 Yakult Central Institute, 5-11 Izumi, Kunitachi, Tokyo 186-8650, Japan; 3 Yakult Honsha European Research Center for Microbiology, ESV, Technologiepark 4, 9052 Ghent-Zwijnaarde, Belgium; 4 Institute of Biochemistry and Molecular Biology, National Yang-Ming University, 155, Sec 2, Li Nong Street, Peitou, Taipei 11221, Taiwan; 5 Faculty of Agricultural Technology and Center for Food & Nutrition Studies, Universitas Gadjah Mada, Bulaksumur, Yogyakarta 55281, Indonesia; 6 Department of Biotechnology, Kasetsart University, 50 Ngam Wong Wan Road, Chatuchak, Bangkok 10900, Thailand; 7 School of Public Health, Faculty of Medicine, Udayana University, Jalan PB.Sudirman, Denpasar 80230, Bali, Indonesia; 8 College of Food Science & Nutritional Engineering, China Agricultural University, 17 Qinghua Donglu, Hai Dian District Beijing 100083, P.R. China; 9 Department of Microbiology, National University of Singapore, 5 Science Drive 2, Singapore 117597, Singapore; 10 Department of Animal Science and Technology, National Taiwan University, 50 Lane 155, Sec 3, Keelung Road, Taipei 10673, Taiwan; 11 Food Industry Research & Development Institute, PO Box 246, Hsinchu 30062, Taiwan; 12 Department of Preventive Medicine, Division of Social Medicine, School of Medical Sciences, Kyushu University, Maidashi 3-1-1, Higashi-ku, Fukuoka 812-8582, Japan; 13 Fermentation Research Center for Value Added Agricultural Products, Khon Kaen University, 123 Mitrapap Road, Amphur Muang, Khon Kaen, 40002, Thailand

## Abstract

Asia differs substantially among and within its regions populated by diverse ethnic groups, which maintain their own respective cultures and dietary habits. To address the diversity in their gut microbiota, we characterized the bacterial community in fecal samples obtained from 303 school-age children living in urban or rural regions in five countries spanning temperate and tropical areas of Asia. The microbiota profiled for the 303 subjects were classified into two enterotype-like clusters, each driven by *Prevotella* (P-type) or *Bifidobacterium*/*Bacteroides* (BB-type), respectively. Majority in China, Japan and Taiwan harbored BB-type, whereas those from Indonesia and Khon Kaen in Thailand mainly harbored P-type. The P-type microbiota was characterized by a more conserved bacterial community sharing a greater number of type-specific phylotypes. Predictive metagenomics suggests higher and lower activity of carbohydrate digestion and bile acid biosynthesis, respectively, in P-type subjects, reflecting their high intake of diets rich in resistant starch. Random-forest analysis classified their fecal species community as mirroring location of resident country, suggesting eco-geographical factors shaping gut microbiota. In particular, children living in Japan harbored a less diversified microbiota with high abundance of *Bifidobacterium* and less number of potentially pathogenic bacteria, which may reflect their living environment and unique diet.

Several hundred microbial species inhabit the human gastrointestinal (GI) tract and constitute a complex ecological community that influences the host's normal physiology and susceptibility to disease through their collective metabolic activities of the community members and their interactions with the host^1^,^2^. Although the gut microbiota varies immensely between individuals, the concept of “enterotypes”’ has been proposed, in which the gut microbial community structures of adult human beings are classified into three types, each defined by the high abundance of *Bacteroides*, *Prevotella*, and *Ruminococcus*^3^. Subsequently, several studies have confirmed the existence of at least two types of microbiomes comprising a preponderance of *Prevotella* or *Bacteroides*, within or across cohorts, although the typing depends on the clustering model used and boundaries between the enterotype clusters become less clear-cut with increase of sample sizes^4^,^5^,^6^,^7^,^8^,^9^,^10^.

Multiple intrinsic and extrinsic factors influence the structure of gastrointestinal (GI)-tract microbiota. Those identified include microbes acquired at birth, diet, host genetics and physiology, drug intake, and disease^11^,^12^,^13^. Diet is considered one of the major drivers for GI-tract microbial community as it provides nutrition and alters the environment for the microbes^14^,^15^,^16^,^17^. Changes in the gut microbiota occur in experimental animals fed high-fat diets that induce obesity^18^,^19^,^20^,^21^. Moreover, a diet comprising a high level of animal proteins, a variety of amino acids, and saturated fats recruits the *Bacteroides* enterotype^15^. Alternatively, controlled diets comprising undigestible carbohydrates provided to overweight men induce significant changes in certain dominant colonic species, although the responses vary among participants^22^.

Cohort studies across distant geographical locations indicate the strong impact of staple foods on the GI-tract microbiota. Compared with children living in Italy, children in rural African village of Burkina Faso harbored GI-tract microbiota that exhibited a unique abundance of bacteria of the genera *Prevotella* and *Xylanibacter* (recently reclassified into *Prevotella*^23^) and a depletion in *Firmicutes*^16^. Similarly, a comparative cohort study on US residents, Amazonas of Venezuela, and rural Malawi showed marked differences in their gut microbiota^6^. It is likely that protein-rich Western diet differentiates their microbiota from those of people who eat carbohydrate-based diet. The influence of regional dietary habits on GI-tract microbiota can be also traced as a signature encoded in microbiome^24^. Genes encoding porphyranase and agarase of *Bacteroides plebeius* were frequently presented in the GI-tract metagenomic data of Japanese population but were absent in the data from North Americans. These carbohydrate-active enzymes digest the polysaccharide porphyran produced by marine red algae, the main seaweed consumed by Japanese, which suggests a link between food and the GI-tract microbiome. These studies indicate that there are global and local variations in the human GI-tract microbiota, which may be attributed to diet and be implicated in host health.

The diets of Asians vary remarkably within the continent and differ significantly from those of other continents. Therefore, our goals were to characterize the gut microbiota of Asians to further understand the correlation between dietary components and the profile of GI-tract microbiota and, ultimately, their link to health and disease. To this end, in the present study, we collected the fecal bacterial composition data of 303 healthy children (at least 25 subjects per city) living in urban and rural cities in China, Japan, Taiwan, Thailand, and Indonesia that span temperate and tropical areas of Asia. Children aged 7 to 11 years old were chosen due to the following two reasons: i) gut microbiota of this age was reported to associate with adult-like configuration deviating from infant microbiota^6^,^25^,^26^ and ii) children of this age mainly eat at home, their diets consist of traditional foods, and their dietary profiles is more uniform and can be accurately tracked. Their fecal microbiota populations were characterized using pyrosequencing and quantitative PCR (qPCR) analysis of bacterial 16S rRNA genes. We address the global and local variations in the fecal bacterial communities of Asian children and discuss the link between these characteristic of Asian microbiota and host factors including dietary habits.

## Results

### Global differences in fecal bacterial community among the Asian children

To investigate the bacterial composition of the GI tracts of healthy Asian children, we collected fresh stool samples from 303 children aged 7 to 11 years old who were living in 10 cities in 5 countries, together with their physiological characteristics, dietary intake, and health condition (Table S1). The V6–V8 region of the 16S rRNA gene was amplified from the stool samples and subjected to the pyrotag sequencing. We acquired 1,704,482 high-quality filtered reads, corresponding to 5,623 ± 2,038 reads per participant. The reads were clustered into 3,003 phylotypes (operational taxonomic units (OTUs) at 97% sequence identity), and their representative sequences were used in taxonomic analysis. The 3,003 phylotypes were classified into known 12 phyla, 56 families, 104 genera, 308 species, and unclassified groups (taxonomic, phylogenetic, and abundance information of the dominant and subdominant phylotypes are described in Supplementary Table S2, Table S3, and Fig. S1).

**Figure 1:**
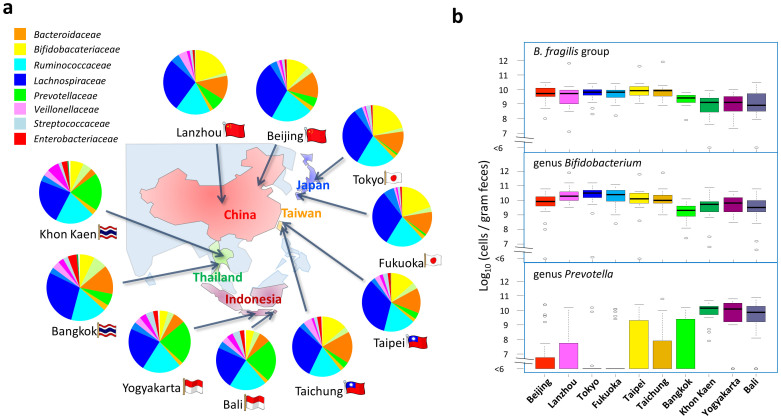
Composition and count of fecal bacteria of the Asian children living in the ten cities. (a) Family-level bacterial composition determined by performing 454-pyrotag sequencing of 16S rRNA genes. Each pie chart represents the mean compositions of the subjects from each city. (b) Cell counts of the *Bacteroides fragilis* group, the genus *Bifidobacterium*, and the genus *Prevotella* were determined using qPCR. For each city, the box plots show the smallest and largest values, 25% and 75% quartiles, the median, and outliers.

The bacterial composition of 303 samples was determined in each taxonomic level according to the read counts of the 3,003 taxonomically-annotated OTUs in each sample. The 303 individual data were statistically compared according to residential place (Supplementary Tables S4 to S7; The effect of age bias is described in “Statistical analysis” in Materials & Methods section). The bacterial composition at family level shows marked differences among the 10 cities (Fig. 1a). The fecal microbiota of children in Khon Kaen, Yogyakarta, and Bali are highly abundant in *Prevotellaceae*, whereas those of children in the other cities are highly abundant in *Bacteroidaceae* and *Bifidobacteriaceae.* These results were further supported by the qPCR data on the number of each bacterial group (Fig. 1b, Supplementary Table S8). In Khon Kaen, Yogyakarta, and Bali, the genus *Prevotella* represented 10^9^–10^10^ cells/g feces, but was not detectable (detection limit approximately 10^6^ cells/g feces) in the majority of children residing in other cities. Notably, in Japan, *Prevotella* was detected in only approximately 10% of the children. In contrast, *Bacteroides fragilis* group and the genus *Bifidobacterium* were detected at >10^8^ cells/g feces in most children (94% and 96%, respectively) residing in the five countries, although they were considerably less populated in Thailand and Indonesia.

### Two enterotype-like clusters identified in Asian children

**Figure 2:**
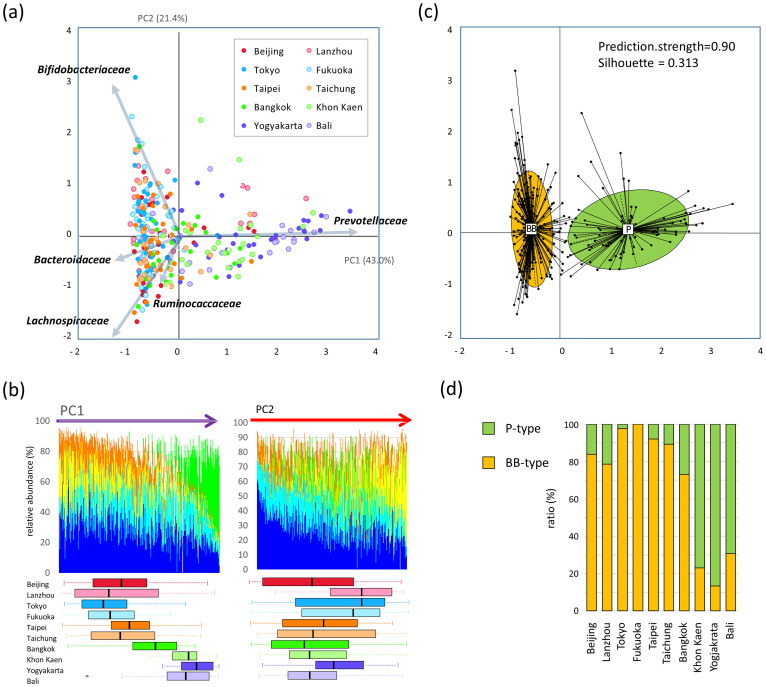
PCA and clustering of samples of 303 Asian children by using species-level composition data. (a) We performed PCA by using the relative abundance data of all bacterial families; in this figure, we have plotted the results according to the PC1 and PC2 scores, with specific colors being used to indicate places of residence. The five largest loadings of bacterial families are indicated by arrows together with their family names. (b) Distribution of the five dominant bacterial families in the 303 Asian children. The bacterial-composition data of the 303 samples were sorted according to the PC1 score (left) and PC2 score (right). In the graphs, the variance between participants from each city is shown in a boxplot. Refer to Fig. 1b for definition of box plot. (c) Clustering of the 303 Asian participants based on family composition data. The 303 samples were clustered using the JSD and PAM clustering. The optimal number of clusters was chosen by maximizing the Calinski–Harabasz index and this was validated based on the prediction strength (PS) and average silhouette width (SW). The clustering is displayed in the PCA plot. The center of gravity of each cluster is indicated by a rectangle filled with the name of microbiota type. The colored ellipse covers 67% of the samples belonging to a cluster. (d) The ratio of the P- and BB-type children in each city.

Using principal component analysis (PCA), family-level bacterial-composition data of all 303 Asian children were decomposed into two factors that explained 64.4% of the variance (Fig. 2a). Principal component 1 (PC1) is heavily loaded with *Prevotellaceae* and negatively loaded with the rest of the predominant families, *Bacteroidaceae*, *Bifidobacteriaceae*, *Lachnospiraceae*, and *Ruminococcaceae*. Principal component 2 (PC2) is heavily loaded with *Bifidobacteriaceae* and negatively loaded with *Lachnospiraceae* and *Ruminococcaceae*. In Fig. 2b, the 303 samples are arranged according to the PC1 score (left panel) and PC2 score (right panel), and the composition of the five largest bacterial families in each sample is graphed. The PC1-ordered graph shows two types of microbiota across the PC1-positive and PC1-negative regions, with each carrying a distinctive core of either *Prevotellaceae* or *Bacteroidaceae*/*Bifidobacteriaceae* along with the common dominant layer of *Lachnospiraceae* and *Ruminococcaceae*. In contrast, the PC2-ordered graph shows a continuous gradient from *Bifidobacteriaceae* to *Lachnospiraceae*. As shown in the box plot under the bacterial composition graph in Fig. 2b, samples from Khon Kaen, Yogyakarta, and Bali are distributed in the PC1-positive region, whereas those from Japan, China, and Taiwan are distributed in the PC1-negative region. For PC2 score, deviation by city of samples is not as obvious as in PC1 but a tendency of high score in samples from Japan and Lanzhou is observed.

Clustering of the 303 Asian samples was attempted on each taxonomic level (Details about the clustering were described in Supplementary Note 1). Significant clustering was achieved and validated from family to phylotype level (Fig. S2, S3, and Table S9). Figure 2c shows the clustering on the PCA at family level. The two clusters are divided into the PC1-positive and PC1-negative regions, which indicate strong reflection of PC1 in the clustering as observed in the PC1 projection shown in Fig. 2b. Henceforth, the *Prevotellaceae*-defined microbiota of PC1-positive group is referred to as “P-type”, whereas the *Bifidobacteriaceae*/*Bacteroidaceae*-defined microbiota of the PC1-negative group is termed as “BB-type”. The ratio of P-type and BB-type in each city is graphed in Fig. 2d. The result is consistent with the data of the PC1 distribution shown in Fig. 2b. Notably, approximately 90% of the samples from Yogyakarta fell into P-enterotype group and all from Japan fell into the BB-enterotype group, except for one samples. The clustering was achieved almost similarly on genus and phylotype level (Fig. S3).

### Community structures of the two types of microbiota

**Figure 3:**
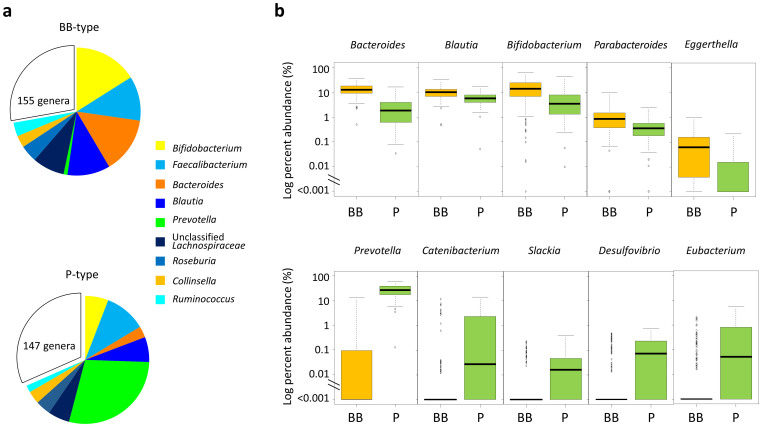
Genus composition of BB- and P-type bacterial communities. (a) Pie charts represent mean relative ratio of the genera in BB- (upper) and P- (lower) type samples, respectively. (b) The box plots represent the relative abundance of the genera enriched in either the BB- (upper) or P- (lower) type. Genera showing the five lowest P values (<10^-9^) in the Mann-Whitney U-test are displayed, respectively. Refer to Fig. 1b for definition of box plot.

Genus compositions of BB- and P-types were compared (Fig. 3). The preponderances of *Prevotella*, *Bifidobacterium*, and *Bacteroides* are confirmed in each type, respectively. Notably, *Prevotella* is depleted from the majority of BB-type subjects while it exists as a most abundant genus in P-type*.* Genus *Blautia* is also significantly enriched in the BB-type and accounts for 10% of total population of BB-type bacterial community, while it accounts for approximately 5% of P-type community. The dominant genus, *Faecalibacterium*, which accounts for more than 10% of total population in our dataset, is evenly distributed between the two types of microbiota. There are some subdominant genera enriched in P-type, e.g., *Catenibacterium*, *Slackia*, *Desulfovibrio*, and *Eubacterium* (lower stage of Fig. 3b). These genera are depleted from majority of BB-type subjects as does *Prevotella*, while the genera enriched in BB-type stay at certain level even in P-type community as shown in upper stage of Fig. 3b.

**Figure 4:**
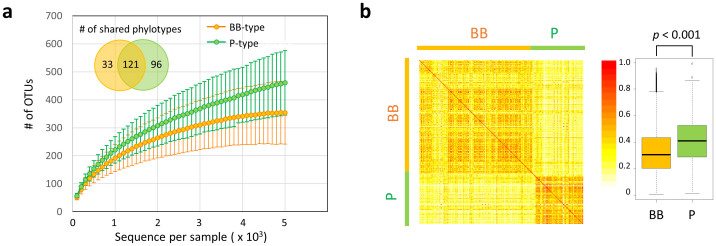
Comparison of alpha- and beta-diversities between BB- and P-type bacterial communities. (a) Rarefaction curve of the number of OTUs observed in subjects for each type. The number of OTUs was determined in each participant at each sampling depth in 100-read increment. The means and standard deviation of the P-type group (n = 88) and the BB-type group (n = 215) are shown in the rarefaction plot. The number of phylotypes shared more than 50% within or across subjects in each group is represented in the inset Venn diagram. (b) Inter-individual similarity of fecal phylotype community between 303 subjects. Pairwise inter-individual similarity was calculated using the Morisita-Horn index and is represented by the heat map and boxplot. Each cell in the heat map, ordered according to BB- and P-type groups, represents the similarity level according to the color scale beside the box plot. Refer to Fig. 1b for definition of box plot.

Community complexities of BB- and P-type microbiota were compared according to alpha- (intra-individual) and beta- (inter-individual) diversities. Rarefaction curves of the number of phylotypes observed in each sample indicates that the P-type individuals harbor a greater number of phylotypes than BB-type subjects (Fig. 4a). The number of common phylotypes shared by >50% of subjects in each group is remarkably higher in P-type than BB-type (Venn graph in inset of Fig. 4a). The distribution of these common phylotypes is displayed in the phylogenetic tree (Supplementary Fig. S4). It indicates that the P-type microbiota largely comprises type-specific phylotypes, in particular, a highly diversified phylogenetic group related to *Prevotella copri.* Also, a number of phylotypes are highly localized in the P-type samples (P < 10^−4^ by chi-square test), some of which are affiliated to the P-type genera shown in Fig. 3b, e.g., *Eubacterium biforme*, *Catenibacterium mitsuokai*, and *Desulfovibrio piger*.

Inter-individual similarity of fecal microbiota was calculated as the Morisita-Horn index and is displayed as a heat map diagram (Fig. 4b). This heat map representation confirms that the BB- and P-type subjects share a similar bacterial community within each group. The similarity, indicated by the heat color in the heat map as well as the accompanying boxplot, is significantly higher in the P-type group than in the BB-type group, suggesting that the P-type community is more conserved among individuals.

### Local variation in fecal bacterial community associated with country

**Figure 5:**
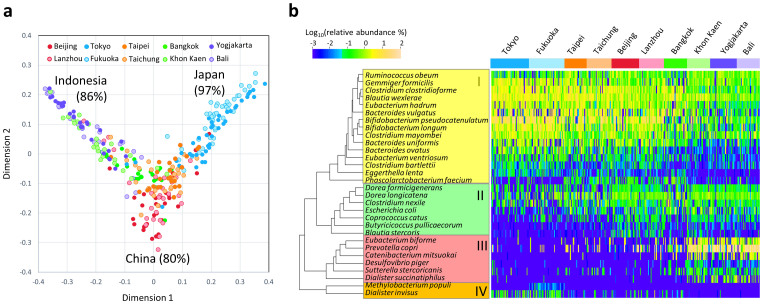
Random forest clustering of 303 Asian children using species composition data. (a) Multidimensional plot of the proximity matrix calculated using random forest analysis of the phylotype composition data of the 303 children. Relative-abundance data of all species in the children were subjected to random forest analysis to perform the machine-learning clustering to identify the country of origin of the samples. The ensemble included 5,000 trees. The calculated proximity matrices are plotted together with the corresponding city colors. (b) Heat map representation of species-level bacterial composition of the microbiota of the 303 Asian children. Top 30 species with the highest Gini score in the random forest analysis performed in (a) were chosen to create the heat map with dendrogram showing the clustering of the species. The relative abundances of these species in each participant were converted to log10 values and subjected to Pearson correlation analysis followed by hierarchical clustering using complete linkage. The population densities of species are scaled by color.

To investigate the local variations in the fecal bacterial community among Asian children rather than the two enterotype-like variations, we employed random forest analysis, which is an ensemble classifier based on a machine-learning algorithm. Fig. 5a shows the result of the random forest classification preformed using species-level composition data of the 303 children; the calculated proximity among the samples is represented in a multidimensional scaling (MDS) plot. Classification was achieved at high probability according to country but not city; the overall out-of-bag (OOB) estimate was 25.7% and 43.9% for country and city, respectively, suggesting the local variation in microbiota more associates with the country of residence. Children living in Japan, China, and Indonesia were classified with high probability, whereas those in Taiwan and Thailand were not clearly differentiated from the others. Interestingly, in the MDS plot, the samples from China form a cluster are localized at the root of two branches extending toward the Indonesian and Japanese clusters, whereas the samples from Thailand and Taiwan are localized between the clusters of China and Indonesia, and China and Japan, respectively.

The abundance of species with the 30 highest Gini scores in the random forest analysis is represented in a heat map (Fig. 5b). The selected 30 species segregate into four clusters according to their relative abundance in the 303 children, as displayed in the column dendrogram. Cluster I mainly comprises the BB-type bacteria such as *Bifidobacterium* and *Bacteroides* species, which are abundant in the six cities from China, Japan, and Taiwan. *Phascolarctobacterium faecium* in Cluster I exhibits a unique distribution profile and is notably abundant in China and Taiwan. Cluster III comprises P-type species such as *P. copri* and *Desulfovibrio piger.* Clusters II and IV display a unique distribution profile independent of the two microbiota types. Cluster II species, including two *Dorea* species, are abundant in China as well as in the P-type cities. Cluster IV species are particularly frequent in Japanese children compared to the other countries. Notably, *Dialister invisus* was detected from 67% of children in Japan but only 18% from other cities. It is known that decrease of *Dialister invisus* is associated with dysbiosis of the fecal microbiota in patients with Crohn's disease^27^.

**Figure 6:**
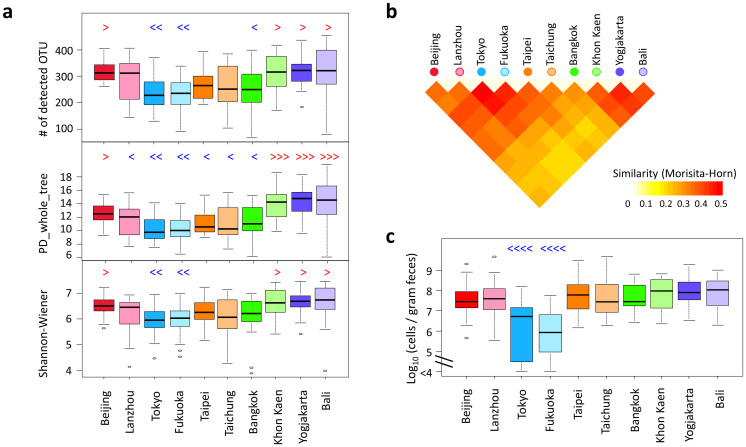
Comparison of fecal microbiota among children in the ten cities. (a) Alpha-diversities of fecal bacterial community in individual samples in each city. Individual phylotype-composition data were rarified using 2,000 reads per participant in two iterations. The number of observed OTUs, PD_whole_tree, and Shannon Wiener index were calculated for each rarified OTU composition and averaged within the two iterations. The covariance of these calculated indices was computed for each country and is graphed as a box plot. (b) The inter-individual similarity indices presented in Fig. 4c were averaged in each block of city pairs and are displayed according to the indicated color scale. (c) Cell numbers of family *Enterobacteriaceae* determined by RT-qPCR. Refer to Fig. 1b for definition of box plot. Red symbols of “>”, “≫”, and “≫>” indicate significantly higher (P < 0.05) than other 2, 4, and 6 cities, respectively, in pairwise Wilcox test; Blue symbols of “<”, “≪”, and“≪≪” indicate significantly lower (P < 0.05) than other 2, 4, and 8 cities, respectively, in the pairwise Wilcoxon rank sum test with Bonferroni correction.

Alpha-diversities of the microbiota were compared among cities (Fig. 6a). The alpha-diversity was evaluated according to the number of detected OTUs, PD_whole_tree, and the Shannon-Wiener index, which were calculated according to the phylotype composition of each sample. Among the ten cities, children from the two cities in Indonesia show the highest diversity, which agrees with the common property of the P-type microbiota. Children from China, particularly those from Beijing, show a high alpha-diversity, even though their microbiota are mostly affiliated to the BB-type. In contrast, the children from the two cities in Japan show particularly low alpha-diversity compared with children from other countries.

The pairwise similarities between samples presented in Fig. 4b were averaged within each pairwise block between cities, and these are represented in the heat map shown in Fig. 6b. Children from Khon Kaen, Yogyakarta, and Bali are highly similar in their fecal bacterial composition, suggesting that they share the conserved P-type bacterial community. Children from the two cities in Japan also shares highly similar fecal microbiota.

Furthermore, a number of interesting features unique to each country or city were observed in the data of their gut bacterial community structure (Table S4 to S8). Details are commented in the Supplementary Note 2. It is noticeable that the level of *Enterobacteriaceae* is remarkably low in children in Japan compared to the other countries' children (Fig. 6c). Although the highly sensitive detection method, reverse-transcription qPCR (RT qPCR), was used in this study, approximately 25% of children in Japan were negative for *Enterobacteriaceae*, while all children in the other countries were positive for this bacteria family.

### Functional properties encoded by the predictive metagenomes of fecal bacterial communities in the Asian children

**Figure 7:**
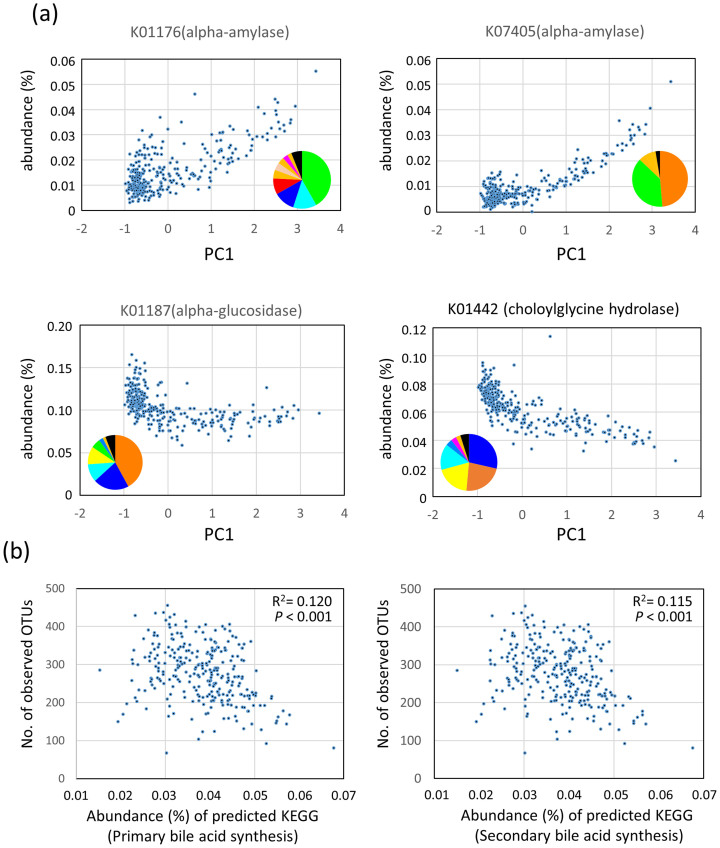
Predictive KEGG functions associated with stool bacterial community structure. (a) Correlation between the abundance of predicted KEGG enzymes and the PC1 score of PCA calculated using the family-level bacterial compositions (see Fig. 2a). Inset pie charts represent the contribution of each bacteria family to this enzyme in our dataset (Refer to Fig. 1a for the color code of bacteria family). (b) Correlation between the abundance of the predicted primary or secondary bile acid synthesis pathway and the number of observed species.

To predict functions encoded by the genomes of bacteria that reside in the guts of Asian children, we performed a phylogenetic investigation of communities by reconstruction of unobserved states (PICRUSt) analysis^28^ based on the 16S rRNA composition data of each subjects. Comparison of the estimated abundances of Kyoto Encyclopedia of Genes and Genomes (KEGG) Orthology groups^29^ between P-type and BB-type samples (Table S10) revealed differences in an orthology group of “carbohydrate digestion and absorption” (*P* = 6.55 × 10^−14^ in Welch *t*-test). Two alpha-amylases (K01176 and K07405) were mainly involved in this orthology group. These two enzyme genes were enriched in the P-type group, although more than 50% of these genes were encoded by non-*Prevotellaceae* family (Fig. 7a). In contrast, an alpha-glucosidase gene (K01187), involved in the digestion of oligosaccharide, was enriched in BB-type samples. The enrichment of amylases and decrease of glucosidase in the P-type bacterial community suggests the higher abundance of undigested carbohydrates in the lower digestive tracts of P-type subjects.

Furthermore, we found a significant difference in the predicted abundance of genes involved in primary and secondary bile acid biosyntheses between P- and BB-types (*P* = 2.98 × 10^−30^ and *P* = 9.81 × 10^−30^, respectively, in Welch *t*-test). The abundance of primary and secondary bile acid syntheses inversely correlated with the number of observed OTUs, which may explain the lower bacterial diversity in BB-type microbiota (Fig. 7b, see “Discussion” for detailed explanation).

### Correlation between dietary habit and fecal microbiota

As shown in the summarized result of food frequency questionnaire (FFQ) (Table S1), rice accounts for the staple carbohydrates of children in the investigated four countries except for children in Lanzhou. The children in China, particularly those in Lanzhou, showed a tendency to depend on wheat as well as rice for their carbohydrate source. This reflects their dietary habit to eat wheat noodles, dumplings, and steamed bread. Children in Indonesia and Thailand eat rice more frequently compared with children living in China and Japan, which agrees with the Helgi Library database (http://www.helgilibrary.com/indicators/index/rice-consumption-per-capita). In particular, most children in Yogyakarta eat rice three times daily. Using the food intake frequency data, we performed logistic regression analysis to correlate diet and the type of gut microbiota (Table S11). The rice-intake frequency significantly correlated with the P-type (Odds ratio = 1.79 with *p* < 0.001). A distinct gradient of rice intake frequency was observed according to PC1, in which subjects who depend heavily on rice are distributed at higher frequency in the P-type cluster of the PC1-positive region (Fig S5). Furthermore, cultivars of rice eaten in each country appear to associate with PC1 (Fig. S5, See Discussion).

Logistic regression analysis also indicated that soybeans, eggs, and chicken correlated positively with P-type (Table S11), whereas a gradient or cluster of their intake frequency was not apparent from the PCA graph (Fig S5), suggesting their influence is not global. P-type children in Indonesia ate soybean more than an average of once a day. Soybeans are eaten commonly as Tempeh in Indonesia. Different from Tofu (soybean curd), Tempeh contains a high concentration of dietary fiber, which may have same effect as the resistant starch derived from rice in the digestive tract (see Discussion)^30^. Eggs and chickens contain high concentration of vitamin A and vitamin B5, which may support the growth of *Prevotella* in intestine as suggested by David et al^31^. Sweet potato, which is one of the highest vitamin A-rich food, is eaten frequently in the P-type country. Indeed, five subjects in Khon Kaen and seven subjects in Yogyakarta ate sweet potatoes every day and all of them harbored the P-type microbiota.

It is noted that the obtained FFQ data do not necessarily explain microbiota of all subjects. For example, P-type subjects in China and Japan did not necessarily follow the P-type carbohydrate-rich diet or the other way around, suggesting that non-investigated factors, maybe including host genetics, interact with gut microbiota. Furthermore, quantitative survey will be required to compare a tremendous variety of Asian diet at nutritional and matrix level. We should also pay attention to the varieties of cultivars and livestock, such as Indica rice and Japonica rice. Cooking method including the amount and type of cooking oils or seasonings, may also be taken into account.

### Correlation between fecal microbiota and host physiology

According to Rohrer index (RI), which represents body leanness-fatness in children, approximately 50% of the subjects in this study were classified in the normal weight and 4.7% and 10.7% were classified in severe underweight and obese, respectively (Fig. S6a). These severe underweight and obese samples are marked in the PCA plot (Fig. S6b). They are uniformally distributed in both P-type and BB-type among the samples from normal weight subjects, suggesting no strong deviation of gut microbiota associated with obesity or leanness. *Bifidobacteriaceae* in BB-type and *Ruminococcaceae* in P-type slightly decreased with increase of RI (Fig. S6c) and negative correlations between RI and *Bifidobacteriaceae* and between RI and *Ruminococcaceae* were observed in BB-type and P-type subjects (Fig. S6d, *R* = − 0.163, *P* = 0.006 for *Bifidobacteriaceae* and *R* = −0.154, *P* = 0.009 for *Ruminococcaceae* in linear regression analysis).

## Discussion

The present study indicates that the variation in the gut microbiota of Asian children clusters into two enterotype-like groups that are driven by trade-off between *Prevotella* and *Bacteroides*/*Bifidobacterium*. This agrees with the findings of a meta-analysis performed using a large Human Microbiome Project (HMP) dataset^7^. The P-type microbiota found in the Asian children is similar in composition to the *Prevotella*-enterotype (ET2) in MetaHIT (Metagenomics of the Human Intestinal Tract) consortium study^3^, while the discrete clusters corresponding to *Bacteroides*-enterotype (ET1) and *Ruminococcus*-enterotype (ET3) observed in the MetaHIT study were not apparent in our samples. Instead, the BB-type cluster diverged widely from the positive to negative range in PC2 with the gradient of the ratio of *Bifidobacteriaceae* to *Clostridiales* (*Lachnospiraceae* and *Ruminococcaceae*). The BB-type cluster appears to contain three subtypes of microbiota, each enriched by *Bifidobacterium*, *Bacteroides*, and *Clostridiales*. The latter two appear to correspond to ET1 and ET3 of the MetaHIT study, respectively^3^, while the *Bifidobacterium*-rich community is unique to our present study.

The high abundance of *Bifidobacterium* is a particular feature of Asian children. The average relative abundance of the genus *Bifidobacterium* was 13% among subjects living in the five countries and 5.5% even among the three P-type cities, whereas none of the data obtained from school-age children in Western cohorts showed a relative abundance of >5% on average for the *Bifidobacterium* population^6^,^16^,^32^. Especially in two cities of Japan and Lanzhou, *Bifidobacterium* accounted for 20%. Interestingly, a study on the diversity of gut microbiota of the Russian population showed that a rural population residing in Tyva in central Asia exhibits a high abundance of *Bifidobacterium* (average approximately 10%), whereas other rural populations, in Tatarstan and Omsk, do not. The carbohydrate-based Asian diet could be a possible factor that drives the colonization of *Bifidobacteria*.

The two microbiota types were strongly associated with country with some exceptions, and were independent of the host physiology represented by Rohrer index. It has been demonstrated that the *Prevotella-*enterotype strongly depends on carbohydrates in diets^15^,^33^ but is robust even during short-term dietary intervention^15^. Further, a recent study on the seasonal dietary changes in the Mongolian population (more animal meat in winter and more dairy products in summer) shows that the abundances of the predominant GI-tract microbiota *Prevotella* and *Bacteroides* remain relatively constant, whereas those of *Faecalibacterium* and *Eubacterium* vary with the seasonal diet^17^. In this study, the rice intake frequency positively correlates with P-type microbiota. However, some Japanese subjects (13%) ate rice three times a day but still had BB-type microbiota, suggesting that other factors associated with the country strongly influence on the microbiota type. One possible explanation is the difference in the cultivar of rice eaten daily in each country (Fig. S5), which differs in the fine structure of starch and influences digestion and absorption in the intestine^34^. The digestion-resistant starch content of rice after cooking using a conventional rice steamer, which is a general practice in these countries, is 6.6% for Indica rice and 0.7% for Japonica rice^35^. This is reflected by the predicted metagenome showing a higher abundance of amylase genes in the P-type bacterial community enriched in Indica/Javanica consumers.

Predictive metagenomics indicated a higher abundance of genes involved in primary and secondary bile acid syntheses in the BB-type bacterial community. This coincides with the finding by David et al.^31^, that the concentration of fecal secondary bile acid increased in response to animal-based diet, which altered gut microbiota composition in 4 days. Conjugated bile acids are secreted from host in response to fat ingestion, deconjugated by choloylglycine hydrolase, and then converted to secondary bile acids^36^. Deconjugated bile acids, particularly secondary bile acids, are rather toxic to intestinal bacteria and host^36^,^37^. The active production of those antimicrobial bile acids predicted in the BB-type bacterial community may hamper the colonization of P-type bacteria and reduce the richness and diversity of resident bacteria^38^. Indeed, *P. copri* and *P. stercorea*, the two dominant *Prevotella* species in the P-type subjects, are sensitive to bile acid^39^. Moreover, the intake of resistant starch decreases the concentration of secondary bile acid^40^,^41^, suggesting the working hypothesis that the resistant starch in rice reaches the lower digestive tract and rescues and/or promote the colonization of P-type bacteria. The link of dietary resistant starch to the P-type microbiota can extend to other crops such as millet and sorghum in Burkina Faso^16^, barley in Mongolia^21^, soybean in Indonesia in this study, but excludes Japonica rice and wheat flour with low contents of resistant starch.

The urbanization trend is seen in the gut microbiota profile of Thai children. The contrast between urban and rural population is remarkable in the distribution of microbiota type in Thailand. Chidren living in Bangkok tended to harbor the BB-type while children living in Khon Kaen children harbor the P-type. The fact that children in Bangkok eat vegetables and fruits much less frequently compared with Khon Kaen children (Table S1) suggests a shift of dietary habits to the modern style in the cosmopolitan city, which may link to the breakdown of the P-type bacterial community. This understanding is supported by our previous study on Thai vegetarians who lived in an urban area but harbored the typical P-type microbiota^42^.

In addition to the enterotype-like global variation, local variation was detected in the fecal microbiomes of Asian children. Random forest analysis presented here highlights the trend in the distribution of Asian microbiota, which diverged from China to Japan and Indonesia, and the gaps between them were filled by samples from Taiwan and Thailand. Interestingly, this trend mirrors the geographical locations linked to the migration of humans as well as agricultural products. In particular, the bacterial composition of children in China and Japan differs remarkably even though they belong to BB-type. The children in Japan harbor a unique microbiota characterized by the high and low abundance of *Bifidobacterium* and *Enterobacteriaceae*, respectively, and low collective and individual diversity. They are particularly less colonized by potentially pathogenic species, such as *Escherichia coli*, *Clostridium perfringens*, and *Lactococcus garvieae* (See Supplementary Note 2). This may be explained by the highly hygienic, modern lifestyle of Japanese citizens in addition to their unique dietary habits. The abundance of *Enterobacteriaceae* is lower in the feces of children in Burkina Faso in rural Africa that correlates with higher concentrations of short chain fatty acid in feces compared to European children^16^. Further, the alpha-diversity of fecal bacterial community in the African children is higher compared with those of European children, suggesting that exposure to environmental microbes may increase the abundance of potentially beneficial bacterial genomes, and a high-fiber diet promotes short chain fatty acid-producing bacteria to prevent establishment of potentially pathogenic intestinal bacteria. This scenario differs from that of the modern social and dietary lifestyles of Japanese children who harbor a microbiota of low diversity. Further studies are therefore warranted to address the question of how a diminished abundance of *Enterobacteriaceae* microbiota is established in Japanese children and how this subsequently affects their health.

In conclusion, the fecal microbiota of the Asian children highly reflects their country of residence, which represents dietary habit and life style. However, hidden factors such as host genetics may interact with their gut microbiota. Further investigation on host factors as well as detail survey on their dietary repertories would contribute to a better understanding of the features of microbial communities in Asian children.

## Methods

### Ethics Statement

This study was approved by the ethics committees of National University of Singapore, Faculty of Agriculture in Kyushu University, Institute for the Development of Human Research Protections, National Yang-Ming University, and Yakult Central Institute. Written informed consent was obtained from the parents/guardians of all participants. We entered and analyzed all samples and questionnaire data anonymously and will publish all data anonymously using personnel numbers. The methods were carried out in accordance with the approved guidelines.

### Sample collection and processing

A total 303 children, aged 7–11-years old, who had not contracted any infectious disease that required medical attention in the two weeks preceding the sampling, provided a single stool sample. All participants were born and grew up in the sampling country. The parents/guardians answered a questionnaire that addressed their physiological characteristics, dietary intake in the past two weeks before stool sampling, and healthy condition. The details regarding these study participants are summarized in Table S1. Fresh feces were collected in separate sterile feces containers (Sarstedt, Nümbrecht, Germany) containing 2 mL of RNA*later* (Ambion, Inc., Austin, TX, USA) and were stored at room temperature. The samples were transferred to the laboratory within 12 h, stored at 4°C, and used for extracting DNA within four weeks. Preliminary experiments confirmed that the bacterial composition data did not change in four weeks under the storage conditions used.

### DNA/RNA extraction

DNA and RNA were extracted from stool samples by using the bead-beating method as previously described^43^, with minor modification. The details are listed in the Supplementary Methods.

### qPCR and RT-qPCR

We performed qPCR to quantify six major bacteria groups^43^ and RT-qPCR to quantify subdominant bacteria groups (Table S8). The detailed protocol is described in the Supplementary Methods. Primers used for the qPCR and RT-qPCR are shown in Table S12.

### 454 pyrotag sequencing and data processing

The V6–V8 region of the bacterial 16S rRNA gene was PCR amplified using the barcode-tag universal primer sets Q-968F-# and Q-1390R-# (where # indicates a series of 128 barcode sequence tags underlined in the sequence)^44^. The forward primer was modified from the prototype primer 968f^45^, which generated less-biased amplification of common GI-tract bacterial members, particularly the *Bacteroidetes*^44^,^46^. The PCR and pyrosequencing were performed as described previously^46^ and the details are listed in the Supplementary Methods. The obtained 454-sequence data comprising 1,866,525 reads were initially processed using the Quantitative Insights Into Microbial Ecology (QIIME) pipeline (http://qiime.org/)^47^ for the purpose of barcode splitting coupled with quality filtering. The resulting set of de-multiplexed sequences was subjected to USEARCH v.5.2.236 to filter out noisy sequences and chimeras and construct OTUs^48^,^49^. Small OTUs comprising fewer than four reads were filtered out in the final step of USEARCH. Consequently, 3,003 OTUs comprising a total of 1,704,482 reads (5,623 ± 2,038 (minimum = 1,043) reads per participant) were obtained as a non-redundant set of OTUs. The read count of each OTU in each sample was tabulated as an OTU table by make_otu_table.py program in the QIIME pipeline and used for the following population analysis. The OTU table was subsampled accordingly to adjust sampling depth of all samples using the multiple_rarefactions.py program in the QIIME pipeline. Details of the data processing are provided in the Supplementary Methods.

### Taxonomic analysis

The taxonomy of each OTU was assigned using the RDP classifier with the confidence threshold of 80% (assign_taxonomy.py) in the QIIME pipeline based on the Greengenes taxonomy (97_otu_taxonomy) and a Greengenes reference database (97_otus.fasta)^50^. To search for closest species, the representative sequence of each OTU was subjected to RDP Seqmatch^51^ in the Ribosomal Database Project II (http://rdp.cme.msu.edu/seqmatch/seqmatch_intro.jsp), in which the lower threshold of the S_ab score was set at 0.84. If more than two species showed the same highest score, the one with the highest count among the top 20 matches was selected for annotating the species by using a Microsoft Excel macro file named Seqmatch Q400^44^. The relative abundance of each taxon was determined by dividing the assigned read counts by the total read counts. The taxonomic and population information of common fecal phylotypes are shown in the Supplementary Table S2 and Table S3.

### Phylogenetic tree analysis

Representative sequences of phylotypes were aligned with an alignment core set of 16S rRNA gene sequences (core_set_aligned.imputed.fasta, Greengene) (http://grenegens.lbl.gov/Download/Sequence_Data/Fasta_data_files/core_set_aligned.fasta.imputed) using the align.seqs program with the suffix-tree search of Mothur software Ver. 1.29.2 (http://www.mothur.org/)^52^. The aligned sequences were filtered using the filter.seqs program in the Mothur package and then processed using the neighbor-joining program in BioEdit Ver. 7.0.9.0 by using DNADIST and NEIGHBOR^53^ from PHYLIP (http://evolution.genetics.washington.edu/phylip.html). The phylogenetic tree was displayed using the iTOL online tool (http://itol.embl.de/)^54^.

### Bacterial composition of each sample

The bacterial composition of each fecal sample was determined at each taxonomic rank according to the OTU table and the taxonomic information of each OTU. Read counts of the OTUs were summed for each taxonomic group by using summarize_taxa_through_plots.py in the QIIME pipeline. The data of the 303 children were compared statistically according to their place of residence (the data are listed in the Supplementary Table S4 to S7).

### Clustering analysis

Clustering was performed according to the enterotyping tutorial provided in the R environment by the EMBL (http://enterotype.embl.de/enterotypes.html). At phylotype level, the OTU table was rarified at 1,000 sequences per sample from five random iterations and the weighted UniFrac distance^55^ between samples was calculated according to the rarified OTU table using the QIIME beta_diversity.py program (http://qiime.org/scripts/beta_diversity.html). At the other taxonomic levels from phylum to genus, the JSD^56^ was calculated according to the relative abundance of each taxon in each sample by using the “dist.JSD” function coded in R (http://enterotype.embl.de/enterotypes.html). Based on the obtained distance matrix, the 303 samples were clustered using PAM clustering by using the “pam” function in the R library “cluster” (R package version 1.14.2, http://cran.r-project.org/web/packages/cluster/index.html). The optimal number of clusters was chosen by maximizing the Calinski–Harabasz index (“index.G1” function in the R library “clusterSim”)(http://cran.r-project.org/web/packages/clusterSim/index.html)^57^. The result of clustering was visualized on PCA plot by the ade4 package in R (http://cran.r-project.org/web/packages/ade4/index.html)^58^. The obtained cluster was validated by the prediction strength (“prediction.strength” function in the R library “fpc”)(http://cran.r-project.org/web/packages/fpc/index.html)^59^ and silhouette index (“silhouette” function in the R library “cluster”)^60^.

### Diversity analysis

The OTU table was rarefied by using the multiple_rarefactions.py script in QIIME (http://qiime.org/scripts/multiple_rarefactions.html). Indices of alpha-diversity including observed_species (the number of OTUs), PD_whole_tree^61^, and Shannon^62^ were calculated for each rarified OTU by using the alpha_diversity.py script in QIIME (http://qiime.org/scripts/alpha_diversity.html). Results from the two random iterations were averaged. The Morisita-Horn index^63^ representing community similarity was calculated by using the SPADE (species prediction and diversity estimation) program (http://chao.stat.nthu.edu.tw/blog/software-download/) and the OTU table rarified to 1,000 reads from five random iterations.

### Random-forest and hierarchical-clustering analyses

Random-forest analysis was performed using the R-package randomForest^64^ (http://cran.r-project.org/web/packages/randomForest/index.html). Calculated proximity matrices were plotted in two dimensions by using the MDSplot function in the randomForest package. Hierarchical analysis was performed using the R-package amap (http://cran.r-project.org/web/packages/amap/index.html). Distances based on Pearson correlations were calculated for input into an agglomerative algorithm through complete linkage, by using the Bioconductor hclust function in the R-package amap. The population densities of species scaled by color are displayed together with dendrogram of bacterial species in a heat map generated using the R-package amap.

### PICRUSt analysis

PICRUSt analysis^28^ was performed by using the online galaxy version (http://huttenhower.sph.harvard.edu/galaxy/root, version 1.0.0). 97% OTUs are picked using a closed-reference OTU picking protocol (QIIME 1.8.0) against the Greengenes database pre-clustered at 97% identify (GG 13.5). The obtained OTU table was normalized by 16S rRNA copy number and metagenomes were predicted from the KEGG catalogue^29^.

### Statistical analysis

To assess the significance of differences between groups, we performed Mann-Whitney U-tests in R 2.13.1 (http://cran.r-project.org) for relative abundance of each bacteria group determined by pyrosequencing of 16S rRNA, Tukey'test in SAS System version 5.0 (Statistical Analysis System Institute Inc.) for the number of each bacterial group determined by qPCR, chi-square test in Excel 2013 (Microsoft Corporation) to examine the localization of phylotypes between groups, and Welch *t*-test in Excel 2013 for relative abundance of each KEGG Orthology group. For comparative analysis of alpha-diversity between cities, pairwise Wilcoxon rank sum test was performed with Bonferroni correction in R 2.13.1. Logistic regression analysis and linear regression analysis were performed in Stata SE12 (Stata Corporation).

To examine confounding effect of age bias among sampling places, we performed multivariate analysis using age and city as the independent variable. The result indicated that the abundance of genus *Bifidobacterium* (-1.75%/y) and two *Lachnospiraceae* genera, *Blautia* (+1.22%/y) and *Roseburia* (+0.72%/y), significantly associated with age but the correlation between bacterial population and sampling city did not changed in the age-adjusted multivariate analysis. Therefore, we concluded that age was not a significant confounding factor to see differences of bacteria population across residential places in our dataset.

## Author Contributions

Conceived and designed the experiments: J.N., K.W., M.C., V.L., C.L., S.N., E.S.R., F.R., Y.T., Y.L. Performed the experiments: J.N., K.W., K.C., J.J., K.M., S.C., P.H., L.O., M.S., I.N.S., L.Z., Y.C., H.C., N.H., C.K., T.K., N.S., K.S., K.T., H.T., T.H. Analyzed the data: J.N., K.W., Y.L. Wrote the paper: J.N., K.W., Y.L.

## Additional Information

**Accession codes:** Sequence data from this article were deposited in the DNA Data Bank of Japan (DDBJ) database under BioProject no. PRJDB1664, which contains links and access to stool sampling data under BioSample SAMD00000022 to SAMD00000324 and pyrotag sequence data designated in the DDBJ sequence read archive (DRA001863 to DRA001872).

## Supplementary Material

Supplementary Information Supplementary Information

Supplementary Information Supplementary Tables S1, S2, S4, S5, S6, S7, S8, S10, S11, S12
