# Author Correction: Diversity in gut bacterial community of school-age children in Asia

**DOI:** 10.1038/s41598-019-42780-z

**Published:** 2019-04-25

**Authors:** Jiro Nakayama, Koichi Watanabe, Jiahui Jiang, Kazunori Matsuda, Shiou-Huei Chao, Pri Haryono, Orawan La-ongkham, Martinus-Agus Sarwoko, I. Nengah Sujaya, Liang Zhao, Kang-Ting Chen, Yen-Po Chen, Hsueh-Hui Chiu, Tomoko Hidaka, Ning-Xin Huang, Chikako Kiyohara, Takashi Kurakawa, Naoshige Sakamoto, Kenji Sonomoto, Kousuke Tashiro, Hirokazu Tsuji, Ming-Ju Chen, Vichai Leelavatcharamas, Chii-Cherng Liao, Sunee Nitisinprasert, Endang S. Rahayu, Fa-Zheng Ren, Ying-Chieh Tsai, Yuan-Kun Lee

**Affiliations:** 1 Department of Bioscience and Biotechnology, Faculty of Agriculture, Kyushu University, 6-10-1 Hakozaki, Higashi-ku, Fukuoka, 812-8581 Japan; 2 Yakult Central Institute, 5-11 Izumi, Kunitachi, Tokyo, 186-8650 Japan; 3 Yakult Honsha European Research Center for Microbiology, ESV, Technologiepark 4, 9052 Ghent-Zwijnaarde, Belgium; 4 Institute of Biochemistry and Molecular Biology, National Yang-Ming University, 155, Sec 2, Li Nong Street, Peitou, Taipei, 11221 Taiwan; 5 Faculty of Agricultural Technology and Center for Food & Nutrition Studies, Universitas Gadjah Mada, Bulaksumur, Yogyakarta, 55281 Indonesia; 6 Department of Biotechnology, Kasetsart University, 50 Ngam Wong Wan Road, Chatuchak, Bangkok, 10900 Thailand; 7 School of Public Health, Faculty of Medicine, Udayana University, Jalan PB.Sudirman, Denpasar, 80230 Bali, Indonesia; 8 College of Food Science & Nutritional Engineering, China Agricultural University, 17 Qinghua Donglu, Hai Dian District, Beijing, 100083 P.R. China; 9 Department of Microbiology, National University of Singapore, 5 Science Drive 2, Singapore, 117597 Singapore; 10 Department of Animal Science and Technology, National Taiwan University, 50 Lane 155, Sec 3, Keelung Road, Taipei, 10673 Taiwan; 11 Food Industry Research & Development Institute, PO Box 246, Hsinchu, 30062 Taiwan; 12 Department of Preventive Medicine, Division of Social Medicine, School of Medical Sciences, Kyushu University, Maidashi 3-1-1, Higashi-ku, Fukuoka, 812-8582 Japan; 13 Fermentation Research Center for Value Added Agricultural Products, Khon Kaen University, 123 Mitrapap Road, Amphur Muang, Khon Kaen, 40002 Thailand

Correction to: *Scientific Reports* 10.1038/srep08397, published online 23 February 2015

The Supplementary Information published with this Article contains errors. In Supplementary Tables S4 and S5, the data in the Yogjakarta columns are duplicates of the data in the Bali columns.

The correct Supplementary Information file is provided below.

## Supplementary information

Supplementary Information

