# Supplementary material for: Diversity in gut bacterial community of school-age children in Asia
Source: Sci Rep. 2015 Feb 23;5:8397. doi: 10.1038/srep08397 (PMC4336934; doi:10.1038/srep08397)
Supplement: Supplementary Information [file srep08397-s1.pdf]

Supplementary Information

Diversity in gut bacterial community of school-age children in Asia

Jiro Nakayama[1#], Koichi Watanabe[2#], Jiahui Jiang[1], Kazunori Matsuda[2,3], Shiou-Huei Chao[4], Pri Haryono[5], Orawan La-ongkham[6], Martinus-Agus Sarwoko[5], I Nengah Sujaya[7], Liang Zhao[8], Kang-Ting Chen[9], Yen-Po Chen[10], Hsueh-Hui Chiu[11], Tomoko Hidaka[1], Ning- Xin Huang[9], Chikako Kiyohara[12], Takashi Kurakawa[2], Naoshige Sakamoto[1], Kenji Sonomoto[1], Kousuke Tashiro[1], Hirokazu Tsuji[2], Ming-Ju Chen[10], Vichai Leelavatcharamas[13], Chii-Cherng Liao[11], Sunee Nitisinprasert[6], Endang S. Rahayu[5], Fa-Zheng Ren[8], Ying-Chieh Tsai[4]*, Yuan-Kun Lee[9]*

[1]Department of Bioscience and Biotechnology, Faculty of Agriculture, Kyushu University, 6-10-1 Hakozaki, Higashi-ku, Fukuoka 812-8581, Japan.
[2]Yakult Central Institute, 1796 Yaho, Kunitachi, Tokyo 186-8659, Japan.
[3]Yakult Honsha European Research Center for Microbiology, ESV, Technologiepark 4, 9052 Ghent-Zwijnaarde, Belgium.
[4]Institute of Biochemistry and Molecular Biology, National Yang-Ming University, 155, Sec 2, Li Nong Street, Peitou, Taipei 11221, Taiwan.
[5]Faculty of Agricultural Technology and Center for Food & Nutrition Studies, Universitas Gadjah Mada, Bulaksumur, Yogyakarta 55281, Indonesia.
[6]Department of Biotechnology, Kasetsart University, 50 Ngam Wong Wan Road, Chatuchak, Bangkok 10900, Thailand.
[7]School of Public Health, Faculty of Medicine, Udayana University, Jalan PB.Sudirman, Denpasar 80230, Bali, Indonesia.
[8]College of Food Science & Nutritional Engineering, China Agricultural University, 17 Qinghua Donglu, Hai Dian District Beijing 100083, P.R. China.
[9]Department of Microbiology, National University of Singapore, 5 Science Drive 2, Singapore 117597, Singapore.
[10]Department of Animal Science and Technology, National Taiwan University, 50 Lane 155, Sec 3, Keelung Road, Taipei 10673, Taiwan.
[11]Food Industry Research & Development Institute, PO Box 246, Hsinchu 30062, Taiwan.
[12]Department of Preventive Medicine, Division of Social Medicine, School of Medical Sciences, Kyushu University, Maidashi 3-1-1, Higashi-ku, Fukuoka 812-8582, Japan.
[13]Fermentation Research Center for Value Added Agricultural Products, Khon Kaen University, 123 Mitrapap Road, Amphur Muang, Khon Kaen, 40002, Thailand.

*Correspondence and requests for materials should be addressed to Y.-K. L. (yuan_kun_lee@nuhs.edu.sg)

Supplementary Methods

1. DNA/RNA extraction

Bacterial DNA was extracted from samples by the bead-beating method and purified as described previously (1), with some modification. In brief, Freshly-voided fecal sample was diluted 10-fold with RNA*later* and homogenized. Then, 200 µl of the fecal sample diluents were mixed with 1 ml PBS and vortexed. After centrifugation at 20,000 × *g* for 5 min at 4 ºC, the supernatant was removed and washed twice with 1 ml of PBS buffer to remove PCR inhibitors. The supernatant was discarded and the pellet was stored at –30 ºC until use. Three hundred milligram of glass beads (diameter, 0.1 mm) (TOMY SEIKO Co., Ltd., Tokyo, Japan), 300 µl of Tris-SDS solution and 500 µl of TE buffer-saturated phenol were added to a thawed sample, and then vortexed vigorously using a FastPrep FP120 (Bio 101) at a speed of 5.0 m/sec for 30 s. Four hundred microliter of phenol/chloroform/isoamyl alcohol (25:24:1; v/v) was added to 400 µl of supernatant and shook vigorously with the use of FastPrep PF120 at a speed of 4.0 m/sec for 45 s. After centrifugation at 20,000 × *g* for 5 min at 4 ºC, 250 µl of supernatant was mixed with 25 µl of 3 M sodium acetate (pH 5.2). After being kept for 3 min on ice, 300 µl of ice cold 100% isopropanol was added and centrifuged at 20,000 × *g* for 5 min at 4 ºC. The pellet of DNA was washed in 500 µl of ice cold 70% ethanol and air dried prior to suspension in 1 ml of TE buffer (pH 8.0) and stored at –30 ºC until use.

RNA was extracted from the stool samples by the method described previously (2). The thawed sample was resuspended in a solution containing 346.5 µl RLT lysis buffer (catalog no. 79216; QIAGEN Sciences, Germantown MD), 3.5 µl β-meracaptoehtanol (Sigma-Aldrich Co., St. Louis, MO) and 100 Tris-EDTA buffer (pH 8.0). Then 300 mg of glass beads (diameter, 0.1 mm; TOMY SEIKO Co., Ltd.) was added to the suspension, and the mixture was vortexed vigorously for 60 s using a FastPrep FP120 (BIO 101) at a speed of 5.0 m/s. Then 500 µl acid phenol (Wako Pure Chemical Industries, Ltd.) was added, and the mixture was incubated at 60 ºC for 10 min. After incubation, the mixture was cooled on ice for 5 min prior to the addition of 100 µl chloroform-isoamyl alcohol. After centrifugation at 12,000 × *g* for 5 min, 400 µl of the supernatant was collected and subjected to isopropanol precipitation. Finally, the nucleic acid fraction was suspended in 50 µl nuclease-free water (Ambion Inc., Austin, TX, USA). To remove contaminating genomic DNA from the RNA fraction, 0.5 U RNase-free DNase I (Takara Bio Inc., Shiga, Japan) per µg RNA was added to each sample in a solution containing 1 µl DNase I buffer (Takara Bio Inc.), followed by incubating at 37 ºC for 20 min. After incubation, the DNase was inactivated and removed twice by acid-phenol and chloroform-isoamyl alcohol extraction as described above, and the RNA in the resultant supernatant was collected by isopropanol precipitation. Finally, the RNA was suspended in 50 µl nuclease-free water.

2. qPCR and RT-qPCR

Quantitative PCR amplification (qPCR) and reverse transcription quantitative PCR (RT-qPCR) were performed in an ABI PRISM 7900HT Sequence Detection system (Applied Biosystems, Foster City, CA, USA). For qPCR amplification, a 10 µl of reaction mixture was composed in 10 mM Tris-HCl (pH 8.3), 50 mM KCl, 1.5 mM $MgCl_2$ 200 µM (each) deoxynucleoside triphosphates, a 1:100,000 dilution of SYBR Green I (Invitrogen), 11 ng of TaqStart antibody (ClonTech, Palo, Alto, CA) per µl, 0.05 U of *Taq* DNA polymerase (Takara Bio) per µl, 0.25 µM (each) specific primers (Table S12), and 1 µl of ×1, ×10, and ×100 diluted temperate DNA. The amplification program consisted of one cycle at 95 °C for 15 min, followed by 45 cycles at 94 °C for 20s, 55 °C for 20 s, and 72 °for 50 s, and finally one cycle of 94 °C for 15 s.

RT-qPCR was carried out using a QIAGEN OneStep RT-PCR kit (QIAGEN, Hilden, Germany). Each 10 µl reaction mixture was composed of 1 × QIAGEN OneStep RT-PCR buffer, 0.5 × Q-solution buffer, each deoxynucleoside triphosphate at a concentration of 400 µM each, a 1:100,000 dilution of SYBR Green I (Invitrogen), 0.4 µl QIAGEN OneStep RT-PCR enzyme mixture, each of the specific primers (Table S12) at a concentration of 0.6 µM, and 5µl template RNA. The reaction mixture was incubated at 50 °C for 30 min for reverse transcription to occur, prior to continuous amplification which consisted of one cycle at 95 °C for 15 min, followed by 45 cycles at 94 °C for 20s, 55 °C for 20 s, and 72 °for 50 s. To distinguish the target PCR product from the non-targeted PCR products, the melt curve was obtained by continuous fluorescence intensity measurements as the reaction mix was slowly heated at temperatures from 60 to 95 °C in increments of 0.2 °C/s. Amplification and detection were carried out in 384-well optical plates with an ABI PRISM 7900HT Sequence Detection system (Applied Biosystems).

3. 454 pyrotag sequencing and data processing

The V6‑V8 region of bacterial 16S rRNA gene was amplified by PCR with a bacterial universal primer set, Q-968F-# (5’-CWSWSWWSHTWACGCGARGAACCTTACC-3’) and Q-1390R-# (5’-CWSWSWWSHTTGACGGGCGGTGWGTAC-3’) (# indicates a series of 128 barcode sequence tags underlined in the sequence) (3). The PCR was performed in a 50 µL volume containing 10 ng to 100 ng extracted DNA as a template, 10 mM Tris-HCl (pH 8.3), 50 mM KCl, 1.5 mM MgCl2, 200 µM deoxynucleoside triphosphate (dNTP) mixture, 10 pmol of each primer and 1.25 U TaKaRa Ex Taq HS (Takara Bio, Otsu, Shiga, Japan). The PCR condition was as follows: 98ºC for 2.5 min; 20 cycles at 98ºC for 15 sec, 54ºC for 30 sec, and 72ºC for 20 sec; and finally 72ºC for 5 min. The PCR products were purified using a QIAquick PCR Purification Kit (Qiagen, Valencia, CA, USA) according to the manufacturer’s protocol. The purified products were quantified using a NanoDrop

ND-1000 microphotometer (NanoDrop Technologies, Wilmington, DE, USA). After that, equal amounts (100 ng) of the amplicons from different samples were pooled and purified prior to pyrosequencing by standard ethanol precipitation. The amplicon mixture DNAs were clonally amplified by emulsion PCR (emPCR) with GS FLX Titanium LV emPCR Kit (Lib-L) v2 according to manufacturer's protocol (454 Life Sciences / Roche Diagnostics). Beads with amplified DNA were loaded onto a GS FLX Titanium PicoTiterPlate with dividers with separate reaction chambers to accommodate two mixture pools. Sequencing was carried out using an FLX Genome Sequencer (454 Life Sciences) with GS FLX Titanium Sequencing Kit XLR70 according to the manufacturer's protocol (454 Life Sciences). In total, pyrosequencing was performed using five half regions for the 303 samples.

The obtained 454 batch sequence data were sorted into each sample batch by using the QIIME split_library.py script (http://qiime.org/scripts/split_libraries.html) with the barcode sequences (4). The parameters used in this script were as follows: l (minimum sequence length) = 408, e (maximum number of errors in barcode) = 0, reverse primer mismatches = 3, a (maximum number of ambiguous bases) = 3, L (maximum sequence length) = 500, s (minimum average quality score allowed in read) = 25. As a result, a total 1,866,525 reads were assigned to the 303 subjects. The quality filtered sequences were subjected to USEARCH ver.5.2.236 (5) installed in the QIIME pipeline to perform filtering of noisy sequences, chimera filtering, and OTU picking. 96,067 sequences were discarded as noisy reads. Chimera filtering was performed though two steps of de novo Uchime and reference-based Uchime in which a template sequence set, gold.fa (http://en.sourceforge.jp/projects/sfnet_microbiomeutil/releases/), was used as reference (6). The denoised reads were de-replicated to unique sequence and was then sorted by abundance. Clusters with less than four reads were discarded and the remaining clusters were subjected to OTU clustering at 97% similarity. As a result, 3,003 OTUs corresponding to 1,704,482 reads [5,623 ± 2,038 (minimum = 1,043) reads per subject] were obtained as non-redundant OTU set. Representative sequence of each OTU was selected by QIIME pick_rep_set.py program using the method "first". Based on the obtained OTU map, the number of times an OTU in each sample was tabulated by make_otu_table.py program in the QIIME pipeline and OTU table was created as biom format. If required, the OTU table was subsampled to adjust sampling depth of all samples by multiple_rarefactions.py program in the QIIME pipeline.

REFERENCES

1. Matsuki, T. *et al*. Use of 16S rRNA gene-targeted group-specific primers for real-time PCR analysis of predominant bacteria in human feces. *Appl. Environ. Microbiol.* **70**, 7220-7228 (2004).

2. Matsuda, K. *et al*. Establishment of an analytical system for the human fecal microbiota, based on reverse transcription-quantitative PCR targeting of multicopy rRNA molecules. *Appl. Environ. Microbiol*. **75**, 1961-1969 (2009).
3. Nakayama, J. Pyrosequence-based 16S rRNA profiling of gastro-intestinal microbiota. *Biosci. Microflora* **29**, 83-96 (2010).
4. Caporaso, J. G. *et al*. QIIME allows analysis of high-throughput community sequencing data. *Nat. Methods* **7**, 335-336 (2010).
5. Edgar, R. C. Search and clustering orders of magnitude faster than BLAST. *Bioinformatics* **26**, 2460-2461 (2010).
6. Edgar, R. C. *et al*. UCHIME improves sensitivity and speed of chimera detection. *Bioinformatics* **27**, 2194-2200 (2011).

Supplementary Note 1

Clustering of fecal bacteria communities of 303 Asian children.

Clustering of the 303 Asian samples was attempted at each taxonomic level. From phylum to species level, clustering was performed using the Jensen-Shannon divergence (JSD) and partitioning around medoid (PAM) algorithm, as performed in the study which originally defined the enterotypes[1]. For phylotype level, the distance was calculated by weighted UniFrac using phylogenetic information[2]. Significant clustering was not observed from phylum to order level, whereas consistent clustering emerged with high significance from family to phylotype level (Table S9). At these taxonomic levels, the optimal number of clusters was suggested to be 2 by maximizing the Calinski–Harabasz (CH) index (inset in Fig. S2) and confirmed by the two indices of prediction strength (PS) and average silhouette width (SW) (Fig. S3a). At the number of cluster equal to two, PS and SW were superior to those of 200 simulation datasets randomized according to the Gaussian deviates based on experimental data (Fig. S3a), suggesting significant clustering that represents two distinct bacteria communities occurring in the gut microbiota of Asian children. The Jaccard coefficient was kept high during 1,000 bootstrap resampling, suggesting the stability of the two clusters (Fig. S3b).

The clusterings on family and genus level are displayed on the PCA plots (Fig. S2a and S2b). The two clusters were divided into the PC1-positive and PC1-negative regions, similarly in both family and genus levels, although the partitioning was somewhat fuzzier in the genus level. Major loadings, namely *Bifidobacteriaceae*/*Bifidobacterium*, *Bacteroidaceae*/*Bacteroides* and *Prevotellaceae*/*Prevotella*, are consistent between family and genus levels, in which *Prevotellaceae*/*Prevotella* drives PC1-positive cluster and the others drive PC1-negative cluster. Loadings corresponding to *Ruminococcaceae* and *Lachnospiraceae* were diminished in the genus level, likely due to the complexity of genus-level taxonomy in these families. The clustering based on the weighted UniFrac distance (Fig. 2c) showed a similar profile to those in family and genus levels; 96% and 91% of samples were consistently classified, respectively. This clustering stayed robust even when whole samples of any one city were removed from the dataset used for clustering (Fig. S3c), suggesting that this clustering does not depend on local variation specific to a certain city but is involved in global distribution of these two microbiota types in the Asian children. Distribution of P- and BB-types of children in each city is nearly consistent among the family, genus, and phylotype level (right panels of Fig. S2).

REFERENCES

1. Arumugam, M. et al., Enterotypes of the human gut microbiome. *Nature* **473** (7346), 174-180 (2011).

2. Lozupone, C. and Knight, R., UniFrac: a new phylogenetic method for comparing microbial communities. *Appl Environ Microbiol* **71** (12), 8228-8235 (2005).

Supplementary Note 2

Features of fecal bacterial community in each country (Data deposited in Table S4 to S8)

1. China

Among BB-type countries, the gut microbiota of children living in Beijing and Lanzhou are characterized by a high abundance of cluster II species, including two *Dorea* species, as indicated by the heat map of Fig. 5b. This feature is shared with P-enterotype cities. Another noticeable common feature in these two Chinese cities is the low abundance of genus *Fusobacterium*, notably species *Fusobacterium mortiferum*. Although the majority of subjects in Beijing and Lanzhou harbored the BB-type microbiota, there are significant differences in their microbiota. The *Bifidobacterium* population represents the major difference between these subjects with averages abundance of 20.0% ($10^{10.25}$ cells/g feces) in Lanzhou and 11.7% ($10^{9.89}$ cells/g feces) in Beijing (P = 0.0043 for cell counts). In contrast, *Lachnospiraceae* and *Ruminococcaceae* are more abundant in children in Beijing than those in Lanzhou. These differences may be due to the unique diet of residents of Lanzhou, where dough noodles are the main source of dietary carbohydrate.

2. Japan

Children in Japan mostly harbored the BB-type microbiota. Among BB-type countries, their microbiota is particularly characteristic in terms of a high abundance of *Bifidobacterium* (20.3%) and relatively low abundance of *Bacteroides* (12.4%), similar to that of the children from Lanzhou. Further, there are a number of distinct features in their microbiota, e.g., a high abundance of families *Peptosterptococcaceae* and *Bacillaceae*, and genera *Veillonella* and *Eggerthella*, and a low abundance of family *Enterobacteriaceae* and genera *Phascolarctobacterium*, *Slakia*, and *Desulfovibrio*. As shown in Fig. 5b, *Dialister invisus* is particularly frequent (67%) in children in Japanese compared to other children (18%). It is known that *Dialister invisus* is associated with dysbiosis of the faecal microbiota in patients with Crohn's disease. It is also interesting that *Bifidobacterium animalis* was detected in 10 out of 83 tested subjects in Japan, while it was detected in only two Chinese subjects in the other countries. *E. coli* (0.10% versus 0.61% for total average) and *Clostridium perfringens* (0.0029% versus 0.088% for total average) were notably less abundant in the children in Japan compared to the other countries. *Lactococcus garvieae* which is known as a fish pathogen was not detected in the children from Japan whereas it was detected in 20% of children from the other countries. The qPCR data also indicates a significantly lower level ($p < 0.01$) and prevalence ($p < 0.05$) of *C. perfringens* and *Enterobacteriaceae* in Japan (Table S8).

It is also evident from Fig. 6a that the gut microbiota of Japanese children is remarkably less diversified compared with children in the other countries. As shown in Fig. 6b, the children from these two Japanese cities possess significantly similar bacteria community. Only minor differences were observed in *Methylobacterium*

populi, which was detected from 85% of tested subjects in Fukuoka (mean abundance = 0.07%), but not detected in children from Tokyo and the other countries.

3. Taiwan

The majority of children in Taiwan harbored the BB-type microbiota. Other than the common features found in BB-type microbiota, there are no distinct features in their bacterial composition. *Bacteroides* population is somewhat higher, especially in Taichung (16.9%) even among BB-type countries.

4. Thailand

BB- and P-types are mixed in Thai children, although there is a tendency that BB- and P-types are enriched in Bangkok and Khon Kaen, respectively. Other than enterotype-like variations, Thai children showed some unique features in their gut bacterial composition. *Enterobacteriaceae* was significantly abundant in children in Thai (2.67%) compared with those in the other countries (1.02%). *L. garvieae*, was also significantly abundant in Thai (0.11%) compared with the other countries (0.026%). There are also features unique to either Bangkok or Khon Kaen, such as a high abundance of *Fusobacteriaceae*, *Collinsella*, and *Phascolarctobacterium* in Bangkok and a high abundance of *Weissella* in Khon Kaen. It is noted that total bacteria count of Bangkok children is significantly lower than the other cities ($p < 0.01$). Further, the abundance of *Bifidobacterium* ($10^{9.1}$ cells /g feces) in children in Bangkok is significantly lower even in the BB-type cities (average $10^{11.2}$ cells / g feces).

5. Indonesia

Children from both cities in Indonesia mainly harbored the P-type microbiota. The abundance of *Prevotella* accounted for 28.7% in Yogyakarta and 22.9% in Bali. Typical of the P-type microbiota, there are a number of families and genera that are markedly abundant in Indonesia, such as *Succinivibrionaceae*, *Victivallaceae*, *Oxalobacteraceae*, *Catenibacterium*, *Slakia*, *Desulfovibrio*, *Succinivibrio*, *Mitsuokella*, and *Comamonas*. It is interesting to note that children in Yogyakarta and Bali shares similar microbiota profiles (Fig. 6b), although their populations have different religious backgrounds that affect their dietary habit as shown in Table S1.

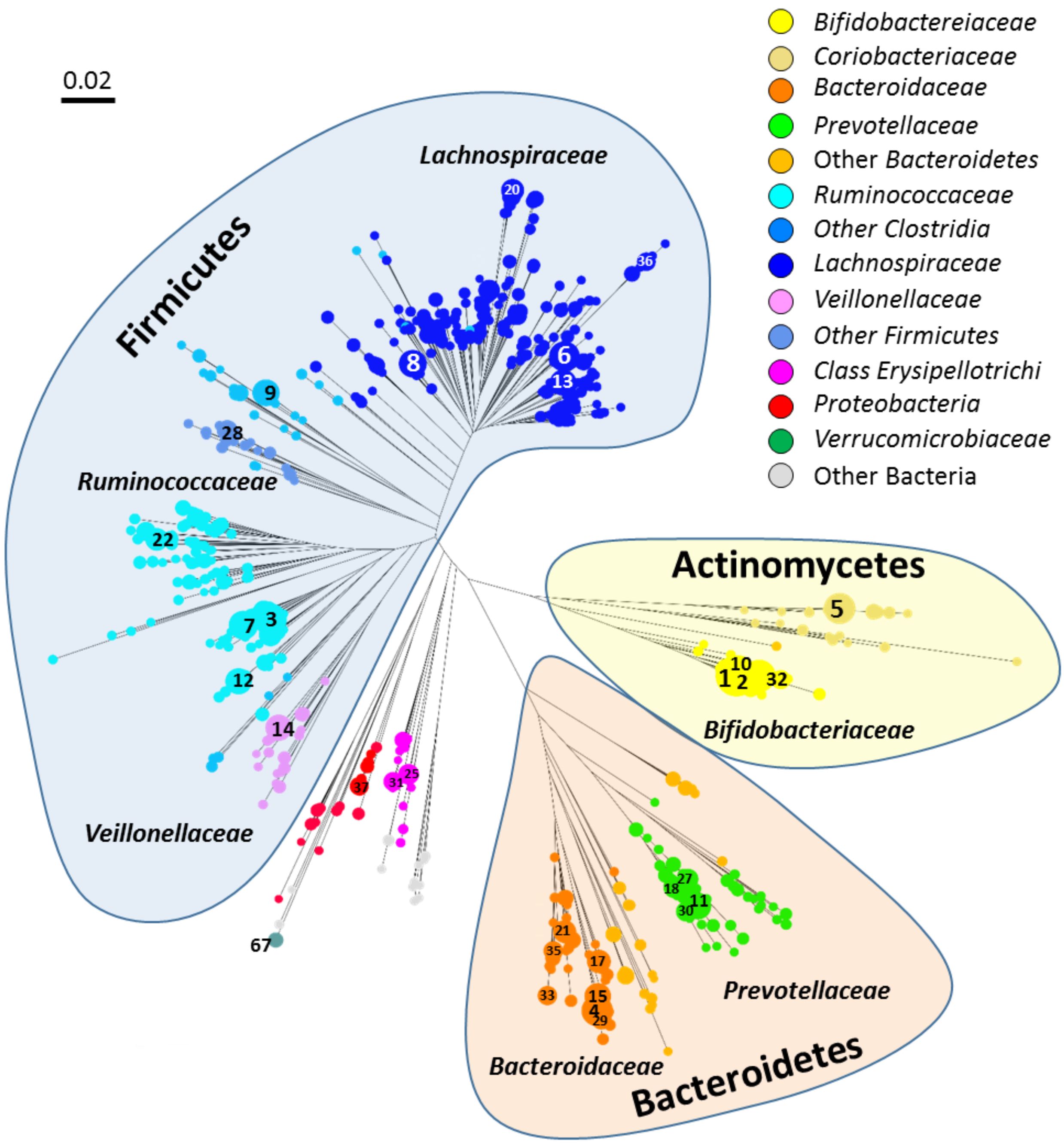

Supplementary Figure S1. Phylogenetic tree of dominant and subdominant phylotypes (overall mean abundance >0.01%) found from stool samples of 303 Asian children. Bubble size and color represent overall abundance in total population and bacterial family of each phylotype, respectively. Dominant phylotypes are labeled with OTU ID (see Supplementary Table S2 and Table S3).

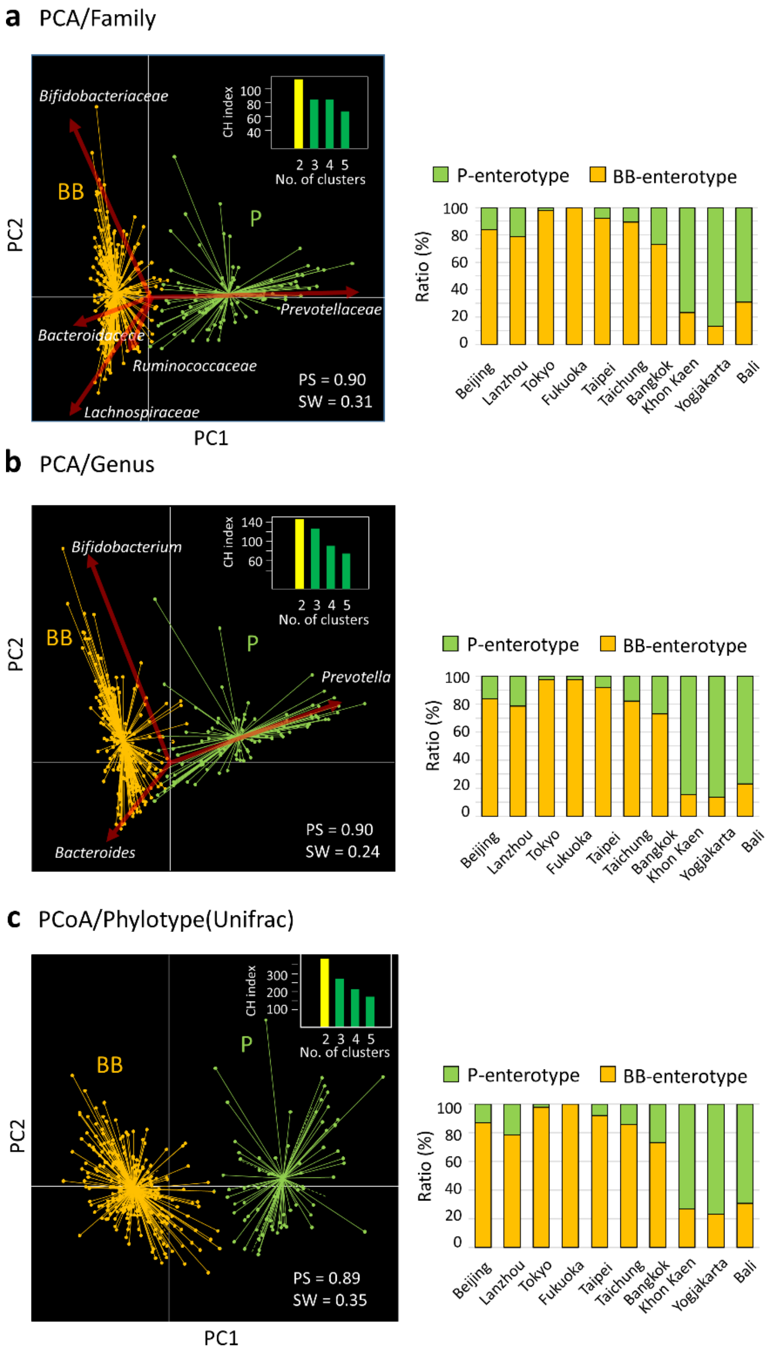

Supplementary Figure S2. Clustering of fecal bacterial communities of 303 Asian children.Clustering of the 303 samples according to bacterial composition data at family (a), genus (b), and phylotype (c) levels. Clustering on family and genus levels was performed using JSD distance and the PAM clustering algorithm. The number of clusters was determined by maximizing the Calinski–Harabasz (CH) index shown in inset graph. The resulting clusters are displayed using the centroid connectors in the PCA plot. Major loading vectors of bacteria are indicated by red arrows. Clustering on phylotype level was performed using weighted-Unifrac distance and the PAM clustering algorithm, and displayed in the principal coordinate analysis (PCoA) plot. The ratio of the P-type and BB-type children in each city was determined in each taxonomic level and was graphed below the PCA or PCoA plot.

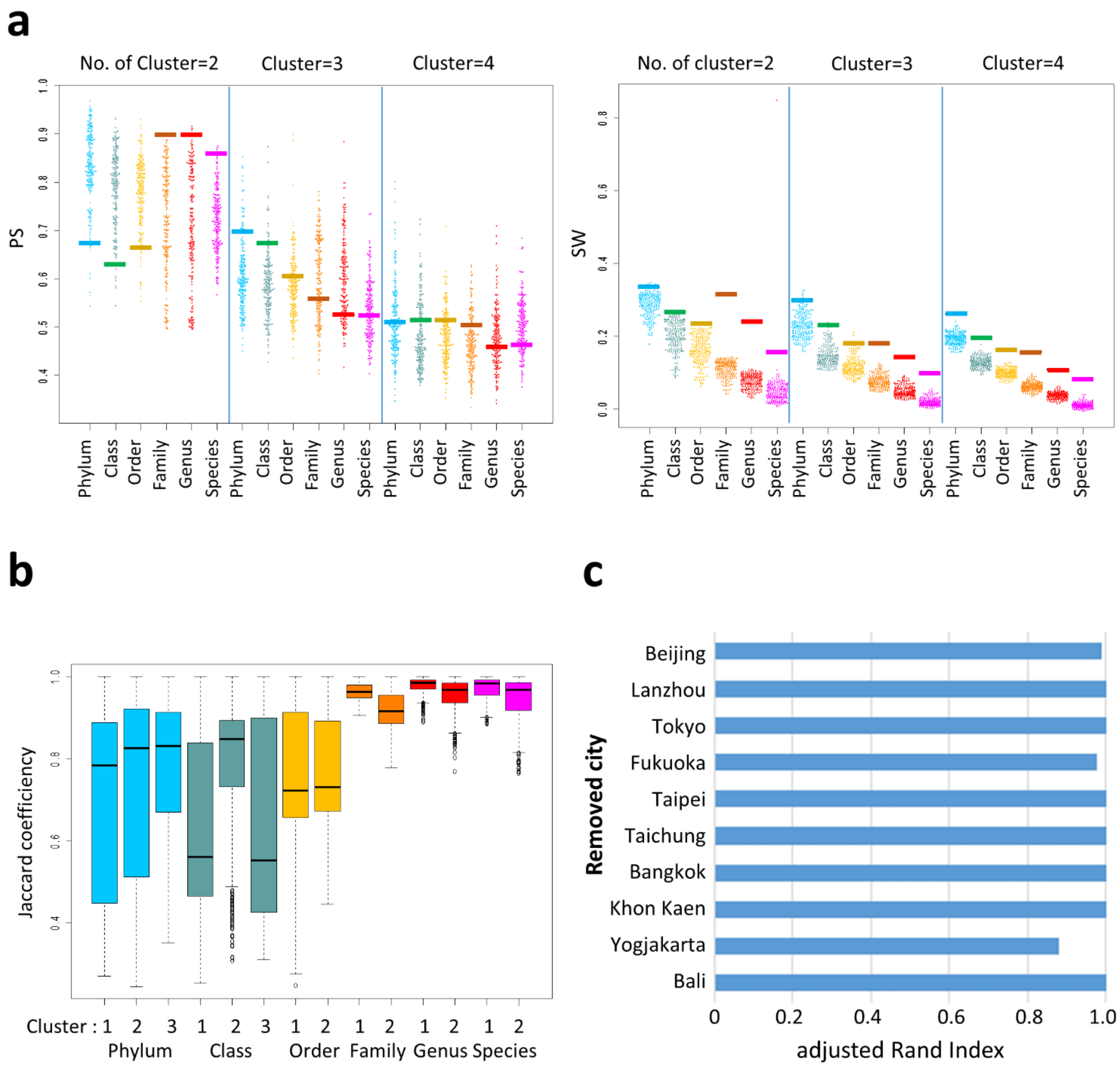

Supplementary Figure S3. Validation of the enterotype-like clustering. (a) Significance of clustering was assessed by comparing prediction strength (PS) scores and average silhouette width (SW) of experimental data (horizontal bar) to 200 simulation data randomized following Gaussian distribution with the same mean and standard deviation as observed in the corresponding experimental data of each taxonomic group (dots). (b) Stability of each simulated cluster was assessed by bootstrap resampling (1,000 times) using “clusterboot” command of the fpc R package in R and displayed by box plots with the smallest and highest values, 25% and 75% percentiles, and the median. (c) Dependency of the type clustering on each city was examined with adjusted Rand index comparing the result of clustering when removing a whole dataset of the city to those obtained from complete dataset. The adjusted Rand index was calculated by using clust.stats program of the fpc R package.

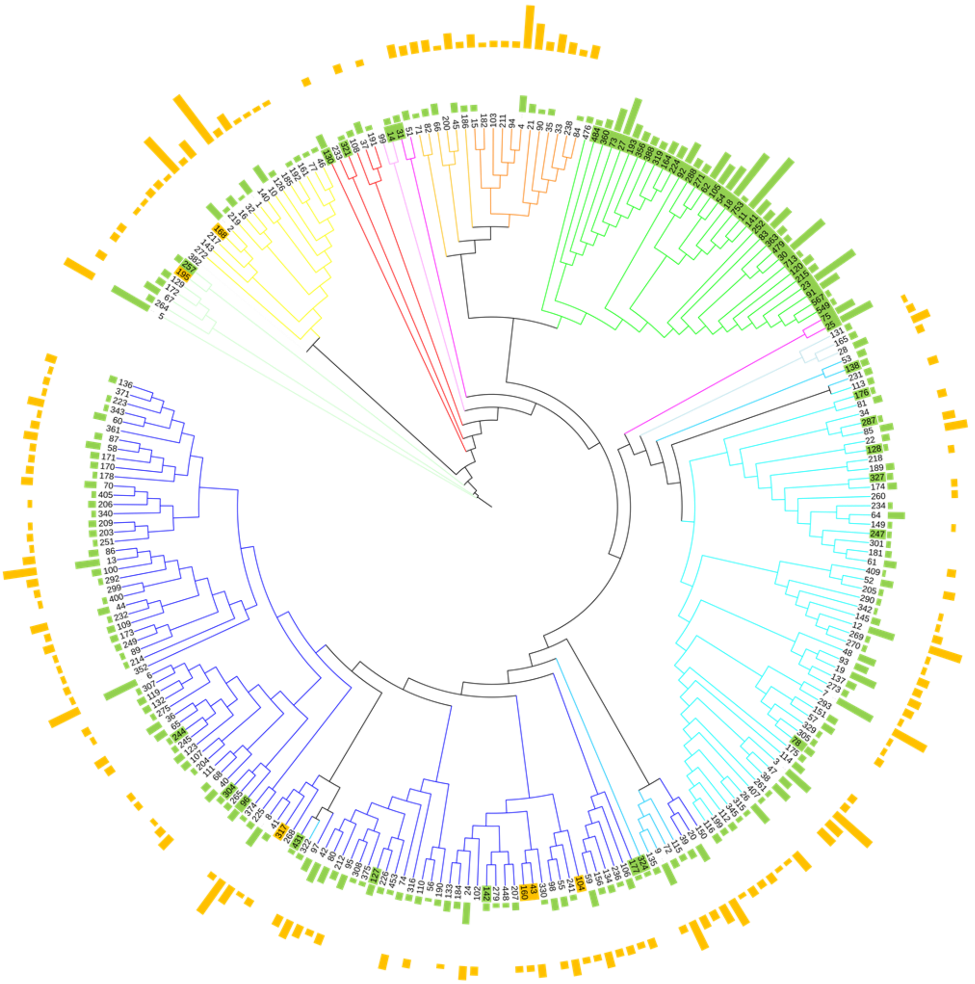

Microbiota-type specific phylotypes
(P < 10^{-4}, Chi-square test)

OUT ID	Type	Closest species
43, 160	BB	*Ruminococcus gnavus*
104	BB	*Robinsoniella peoriensis*
168	BB	*Bifidobacterium breve*
195	BB	*Eggerthella lenta*
317	BB	*Clostridium clostridioforme*
14	P	*Megamonas funiformis*
23*	P	*Prevotella copri*
25,75	P	*Eubacterium biforme*
31	P	*Catenibacterium mitsuokai*
78	P	*Faecalibacterium prausnitzii*
96	P	*Bacteroides xylanolyticus*
127	P	*Clostridium nexile*
128	P	*Eubacterium coprostanoligenes*
130	P	*Sutterella stercoricanis*
138	P	*Clostridium chartatabidum*
142	P	*Ruminococcus lactaris*
176	P	*Ruminococcus callidus*
177	P	*Clostridium scindens*
244	P	*Roseburia faecis*
257	P	*Adlercreutzia equolifaciens*
287	P	*Butyricicoccus pullicaecorum*
304	P	*Eubacterium ramulus*
321	P	*Desulfovibrio piger*
324	P	*Clostridium lactatifermentans*
327	P	*Oscillibacter valericigenes*
431	P	*Clostridium hathewayi*

*Only one representative ID are shown.

Supplementary Figure S4. Phylogenetic tree of 250 common phylotypes. Phylotypes observed in >50% of subjects in each type group were chosen (see details in Supplementary Table S2) and their sequences were subjected to the phylogenetic analysis. The median of their abundances in the P- and BB-type groups is represented by the bar chart outside the tree. Phylotypes showing P value < 10^{-4} in the chi-square test between P- and BB-type groups are colored on their ID no. and are listed in the right-side table with their closest species name. The colors used for the tree branches represent bacterial families (see Fig. 1b).

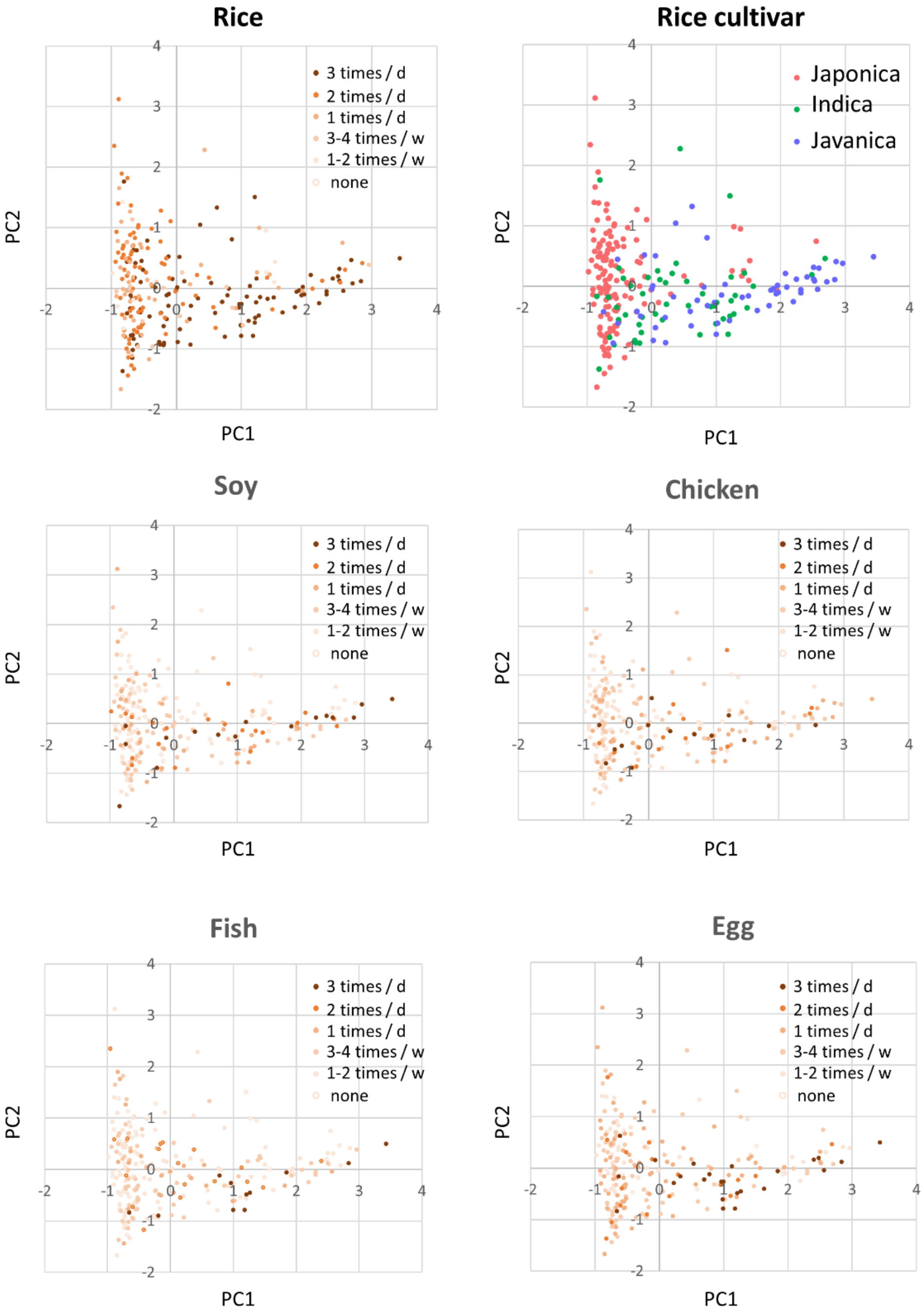

Supplementary Figure S5. Superimpose of food frequency and rice cultivar on the PCA calculated using family-level bacteria composition (See Fig. 2a). Food frequency data was obtained by the questionnaire asking intake frequency of the corresponding diet in the past two weeks before stool sampling. Rice type is investigated due to inquiry survey to the co-investigator of each country in this project. Generally, children in China and Japan mostly eat Japonica rice, those in Thailand eat Indica rice, and those in Yogjakarta and Bali eat Javanica rice.

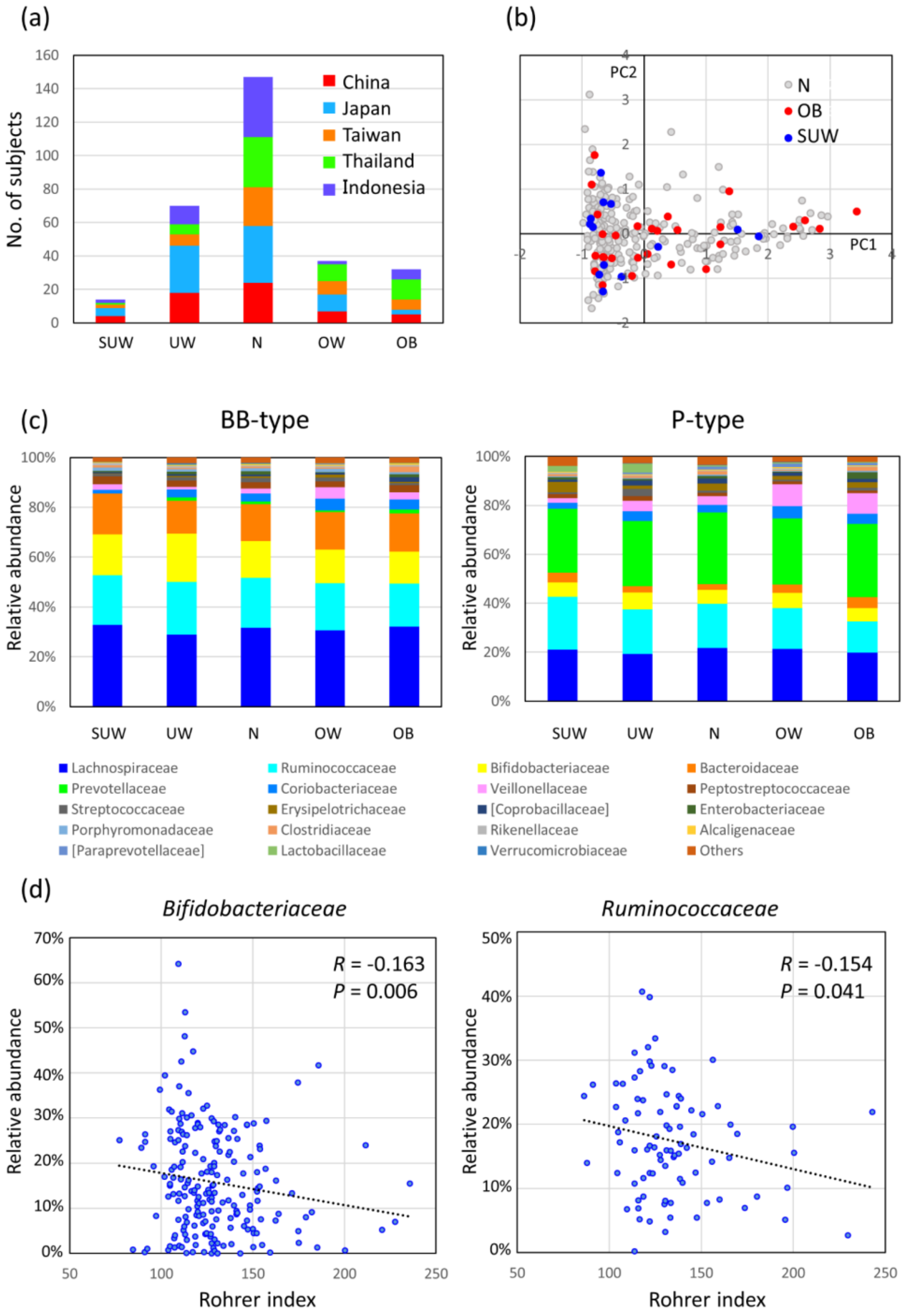

Supplementary Figure 6. Leanness/fatness of subjects in this study and their correlation with fecal microbiota. (a) Distribution of subjects classified according to Rohrer index (RI); SUW (severe underweight, RI less than 100), UW (underweight, RI between 100 and 115), N (Normal, RI between 115 and 145), OW (overweight, RI between 145 and 160), and OB (obese, RI over 160). (b) SUW and OB subjects on the PCA plot based on fecal microbiota at family level (See Fig. 2a). (c) Family-level bacteria composition of stool samples from different RI groups. (d) Correlation between RI and relative abundance of *Bifidobacteriaceae* or *Ruminococcaceae*. Correlation significance is represented as *R* and *P* values in linear regression analysis.

Supplementary Table S3. Prevalence and abundance of dominant and subdominant phylotypes observed in the 303 Asian children.

OTU ID[a]	Closest species (similarity)[b]	Carrier #[c] (0 100 200 300)	Mean by country (%)[d] China	Japan	Taiwan	Thailand	Indonesia
1	*Bifidobacterium pseudocatenulatum* (1.000)		6.31	8.40	4.16	1.40	0.43
2	*Bifidobacterium longum* (1.000)		2.46	4.52	2.51	1.70	0.55
3	*Faecalibacterium prausnitzii* (0.997)		1.95	3.69	2.84	1.90	1.22
4	*Bacteroides vulgatus* (0.997)		2.06	2.85	3.42	2.40	0.59
5	*Collinsella aerofaciens* (0.995)		1.69	1.69	2.27	3.70	2.44
6	*Eubacterium rectale* (0.997)		2.68	2.60	1.34	1.47	2.41
7	*Faecalibacterium prausnitzii* (0.987)		1.69	1.83	1.85	1.94	1.90
8	*Clostridium clostridioforme* (1.000)		1.67	2.31	2.35	1.22	0.57
9	*Clostridium mayombei* (0.997)		1.71	2.53	1.25	0.70	1.22
10	*Bifidobacterium adolescentis* (0.997)		1.62	1.88	2.08	0.66	1.09
11	*Prevotella copri* (0.966)		1.15	0.17	0.36	1.82	4.64
12	*Gemmiger formicilis* (0.990)		0.99	2.23	1.33	1.49	0.80
13	*Blautia wexlerae* (0.992)		0.97	1.88	1.86	1.69	0.57
14	*Megamonas funiformis* (0.997)		1.69	0.30	2.31	1.95	1.35
15	*Bacteroides dorei* (0.997)		2.06	2.04	1.59	0.49	0.36
17	*Bacteroides plebeius* (0.997)		1.57	0.54	2.37	0.63	0.39
18	*Prevotella copri* (0.984)		1.02	0.16	0.59	1.31	2.42
20	*Eubacterium hadrum* (0.992)		1.07	1.12	1.42	0.90	0.29
21	*Bacteroides uniformis* (0.997)		1.43	1.23	1.07	0.59	0.27
22	*Ruminococcus bromii* (1.000)		1.14	1.61	0.71	0.45	0.44
25	*Eubacterium biforme* (1.000)		0.30	0.08	0.55	1.68	1.53
27	*Prevotella copri* (0.989)		0.19	0.00	0.06	1.03	2.58
28	*Streptococcus thermophilus* (1.000)		0.66	0.73	0.94	0.73	0.44
29	*Bacteroides coprocola* (0.997)		1.37	0.40	0.90	0.41	0.47
30	*Prevotella copri* (0.989)		0.58	0.08	0.28	0.83	1.69
31	*Catenibacterium mitsuokai* (0.997)		0.20	0.00	0.08	1.53	1.67
32	*Bifidobacterium bifidum* (1.000)		0.80	0.72	0.19	0.68	0.54
33	*Bacteroides fragilis* (0.997)		0.63	0.53	0.37	0.73	0.63
34	*Ruminococcus albus* (0.956)		0.68	0.96	0.62	0.13	0.25
35	*Bacteroides ovatus* (0.995)		0.42	1.11	0.83	0.10	0.13
36	*Roseburia faecis* (0.997)		0.48	0.57	0.82	0.30	0.49
37	*Escherichia coli* (0.995)		0.37	0.09	0.69	1.28	0.52
76	*Akkermansia muciniphila* (1.000)		0.17	0.12	0.13	0.71	0.28

[a]Common ID No. also used in Supplementary Table S2, Fig. S1 and Fig. S4. Coloured circle represents bacteria family (see the index in Supplementary Fig. S1).

[b]Closest species were searched by RDP seqmatch followed by Seqmatch Q400 algorithm. The values in parentheses represent sequence identity to the 16S rRNA of the indicated species in the RDP-II database.

[c]The number of carriers is indicated by the green bar graph.

[d]Mean abundance (%) by country is shown and coloured by red according to the abundance.

Table S9. Cluster analysis at each taxonomic rank.

Taxonomic rank	Cluster number[a]	PS/SW[b]	Cluster size[c]
Phylum	3	0.70/0.30	130/72/101
Class	3	0.69/0.23	117/84/122
Order	2	0.68/0.23	191/122
Family	2	0.90/0.31	215/88
Genus	2	0.90/0.24	208/95
Species	2	0.86/0.16	213/90
Phylotype[d]	2	0.89/0.35	219/84

[a]The optimal number of clusters was chosen by maximizing the Calinski–Harabasz index.
[b]PS/SW: Prediction strength/average Silhouette width.
[c]The number of samples assigned to each cluster.
[d]Weighted UniFrac was used to calculate distance, while JSD was used for other taxonomic levels.
